# The Nucleocapsid Region of HIV-1 Gag Cooperates with the PTAP and LYPX_n_L Late Domains to Recruit the Cellular Machinery Necessary for Viral Budding

**DOI:** 10.1371/journal.ppat.1000339

**Published:** 2009-03-13

**Authors:** Vincent Dussupt, Melodi P. Javid, Georges Abou-Jaoudé, Joshua A. Jadwin, Jason de La Cruz, Kunio Nagashima, Fadila Bouamr

**Affiliations:** 1 Laboratory of Molecular Microbiology, National Institute of Allergy and Infectious Diseases, National Institutes of Health, Bethesda, Maryland, United States of America; 2 SAIC at NCI-Frederick, Frederick, Maryland, United States of America; King's College London School of Medicine, United Kingdom

## Abstract

HIV-1 release is mediated through two motifs in the p6 region of Gag, PTAP and LYPX_n_L, which recruit cellular proteins Tsg101 and Alix, respectively. The Nucleocapsid region of Gag (NC), which binds the Bro1 domain of Alix, also plays an important role in HIV-1 release, but the underlying mechanism remains unclear. Here we show that the first 202 residues of the Bro1 domain (Bro_i_) are sufficient to bind Gag. Bro_i_ interferes with HIV-1 release in an NC–dependent manner and arrests viral budding at the plasma membrane. Similar interrupted budding structures are seen following over-expression of a fragment containing Bro1 with the adjacent V domain (Bro1-V). Although only Bro1-V contains binding determinants for CHMP4, both Bro_i_ and Bro1-V inhibited release via both the PTAP/Tsg101 and the LYPX_n_L/Alix pathways, suggesting that they interfere with a key step in HIV-1 release. Remarkably, we found that over-expression of Bro1 rescued the release of HIV-1 lacking both L domains. This rescue required the N-terminal region of the NC domain in Gag and the CHMP4 binding site in Bro1. Interestingly, release defects due to mutations in NC that prevented Bro1 mediated rescue of virus egress were rescued by providing a link to the ESCRT machinery via Nedd4.2s over-expression. Our data support a model in which NC cooperates with PTAP in the recruitment of cellular proteins necessary for its L domain activity and binds the Bro1–CHMP4 complex required for LYPX_n_L–mediated budding.

## Author Summary

Human immunodeficiency virus type 1 (HIV-1) assembles its structural protein Gag into a viral shell at the plasma membrane. Gag is divided into several regions, each with its own distinct function(s). Within the p6 region of Gag, there are two short peptide sequences, called Late (L) domains, that serve to recruit cellular proteins Tsg101 and Alix. In an uninfected cell, these proteins facilitate membrane dynamics during vesicle budding into cellular compartments called endosomes. Upon infection, HIV-1 hijacks these proteins and makes use of the machinery to facilitate viral budding at the plasma membrane. Our study shows that, in addition to binding the p6 region, Alix also interacts with the Nucleocapsid (NC) region of Gag. Importantly, we show that when HIV-1 buds via the Alix-driven pathway, this interaction with NC is essential for recruiting host proteins necessary for HIV-1 release. Moreover, we show that a non-functional fragment of Alix inhibits Tsg101-mediated HIV-1 release in ways similar to those caused by mutations in the NC domain of Gag. Collectively, our findings favor a model in which the p6-located L domain motifs require cooperation with NC to facilitate HIV-1 release.

## Introduction

The human immunodeficiency virus type I (HIV-1) Gag polyprotein, p55Gag, is the main structural component of viral particles [1]. It carries four distinct domains: the N-terminal Matrix (MA), the central capsid (CA), the Nucleocapsid (NC) and the C-terminal p6 region. MA is responsible for targeting Gag to the plasma membrane for assembly *via* a bipartite signal composed of a myristic acid moiety and a cluster of basic residues [2],[3],[4]. The CA domain bears regions essential for Gag-Gag multimerization and is the main constituent of the viral core [5],[6]. The NC domain promotes Gag-Gag assembly via its ability to interact with RNA [7],[8],[9]. Viral particle budding from the plasma membrane requires the activity of L domain motifs within p6 [10],[11], which recruit cellular proteins necessary for membrane fission and release [12],[13],[14],[15].

Two late domains have been identified within the p6 of HIV-1 Gag, the PTAP and LYPX_n_L motifs. The PTAP motif binds the cellular protein Tsg101 [16],[17],[18] whereas the LYPX_n_L motif is the docking site for Alix/AIP-1 [19],[20],[21]. Tsg101 functions in HIV-1 budding [16],[22],[23] as a member of the Endosomal Sorting Complex Required for Transport-1 (ESCRT-I) [21],[24],[25], which initiates the sorting of surface proteins into late endosomal compartments known as *m*ulti*v*esicular *b*odies (MVB) [26],[27],[28]. This process is topologically similar to HIV-1 budding and requires the recruitment of ESCRT-III members called the *ch*arged-*m*ultivesicular body *p*roteins (CHMP) [19],[20],[21],[29] and the activity of VPS4 AAA-type ATPase [21],[30],[31],[32],[33],[34]. The PTAP/Tsg101 driven pathway is considered to be the predominant mechanism for HIV-1 release.

The second L domain motif, LYPX_n_L, binds Alix [19],[20],[21],[35],[36],[37], an ALG-2 interacting host protein [38],[39] presumed to function in endosomal metabolism [40],[41],[42]. Structural studies revealed that Alix is comprised of three distinct domains, an N-terminal Bro1 domain, a LYPX_n_L binding coiled-coil V domain, and a C-terminal *p*roline *r*ich *d*omain (PRD), which interacts with several cellular factors including Tsg101 [19],[41],[43],[44],[45]. The N-terminal Bro1 domain binds the ESCRT-III CHMP4 proteins [44],[46],[47]. The banana shaped structure of the Bro1 domain [44],[46] suggests a possible role in membrane curvature [48] during the normal function of Alix in both MVB and retroviral budding. Although Alix is currently considered to play a supporting role in HIV-1 release, it was recently shown to facilitate HIV-1 release in absence of the PTAP motif [44],[47]. Furthermore, Alix is sufficient for release of *E*quine *I*nfectious *A*nemia *V*irus (EIAV), which contains only the LYPX_n_L type L domain motif [20],[44],[47],[49].

The ESCRT-III CHMP proteins are recruited by HIV-1 Gag either indirectly, through the Tsg101-PTAP motif interaction, or directly by the V domain of Alix binding to the LYPX_n_L motif [19],[20],[21]. Recent studies showed that recruitment of CHMP4 proteins by Bro1 is required for Alix function in HIV-1 release [44],[47]. Most recently, it was shown that CHMP4 proteins self-associate into filaments that promote membrane bending away from the cytoplasm [50]. Interestingly, over-expression of dominant negative versions of CHMP proteins induces Class E-like phenotypes and inhibits HIV-1 budding [19],[20],[21],[51],[52],[53]. These studies are consistent with a role for ESCRT-III members, especially the CHMP4b isoform, in facilitating fission events that lead to HIV-1 separation from the plasma membrane.

In recent years, multiple studies have suggested that the ability of L domain motifs to facilitate budding and release is context-dependent [35],[54],[55],[56],[57]. This implies that they cooperate with other regions within Gag to achieve efficient viral release [35],[55],[56],[57],[58],[59]. Consistent with this notion, the PTAP motif was shown to cooperate with the NC-p1 regions [57] and also to require p6 residues 15–51, which include the Alix binding site, to function optimally [55]. Most recently, the NC domain of Gag (hereafter also called NC) was found to engage the Bro1 domain of Alix, an interaction that is required for the LYPX_n_L/Alix pathway to function in HIV-1 release [60]. Mutations within the NC domain or deletion of the entire domain significantly reduce the release of HIV-1 particles even though the NC-deleted Gag retains the ability to assemble particles [56],[61],[62],[63],[64]. Together these observations suggest a role for the NC domain in HIV-1 budding driven by L domain motifs located within the neighboring p6 domain. The temporal and spatial involvement of NC in particle budding and egress, and the extent to which it functions with p6 have not yet been clearly established.

The absence of direct evidence for an NC role in HIV-1 budding, and of effective means of interfering with such a function, make it difficult to establish a functional link between p6 Late domain motifs and NC. We used Alix to examine a possible role for NC in HIV-1 budding and identified a small fragment of Bro1 that inhibits both Tsg101/PTAP and Alix/LYPX_n_L budding pathways in an NC-dependent manner. Over-expression of the full length Bro1 domain rescues budding defects caused by disruption of the PTAP and LYPX_n_L motifs; rescue requires a CHMP4 binding site in Bro1 and an intact NC. Mutations in NC that prevent Bro1-mediated viral release also inhibit the release of a PTAP-dependent HIV-1 suggesting that NC function is required for both pathways. Interestingly, mutants defective due to mutations in NC can be rescued by providing a link to ESCRT components via a parallel pathway. Collectively, our results support a model in which NC cooperates with the PTAP and LYPX_n_L motifs in p6 to recruit components of the cellular machinery necessary for HIV-1 budding.

## Results

### Over-expression of Alix inhibits HIV-1 budding

**Figure 1:**
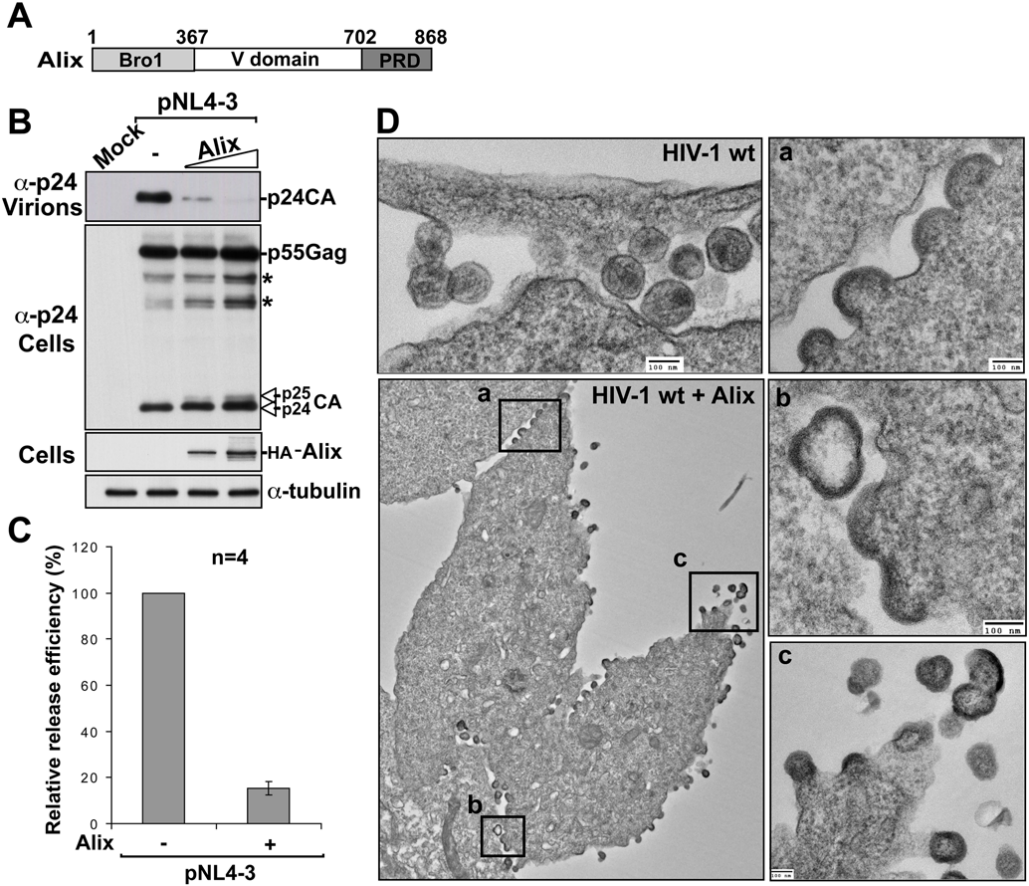
Over-expression of Alix inhibits HIV-1 release. (A) Schematic representation of the domain organization of Alix. Bro1: *B*CK1-like *r*esistance to *o*smotic shock *1*, V: V-shaped domain, PRD: *P*roline *R*ich *D*omain. Numbers indicate positions of amino acid residues. (B) Virus-release assay showing that over-expression of Alix inhibits virus release. 293T cells were transfected with either wild-type (wt) pNL4-3 plasmid alone or with increasing amounts of HA-Alix. Pelleted virions and cell lysates were analyzed by SDS-PAGE and western blot using the indicated antibodies. Gag intermediate products are indicated with (*) symbols. (C) Relative release efficiency of HIV-1 virions upon over-expression of Alix. The release efficiency in presence of Alix (calculated at the highest amount shown in B, lane 4) is relative to the release efficiency of NL4-3 alone, which was arbitrarily set at 100. Error bars indicate the standard deviation from four separate experiments. (D) Transmission Electron Microscopy (TEM) images of thin-sectioned 293T cells transfected with pNL4-3 wt alone (upper left panel) or pNL4-3 wt + Alix (lower left panel and close-ups of regions of interest a, b, c) showing arrested budding particles.

HIV-1 recruits Tsg101 and Alix to bud from the plasma membrane of infected cells. When the Tsg101-driven pathway is not available (HIV-1 PTAP- mutant), over-expression of Alix has been shown to rescue HIV-1 release defects [44],[47]. Titration analysis indicated that the optimal amount of HA-Alix that stimulated HIV-1 PTAP- was relatively low (Figure S1A, lane 3), whereas in contrast, higher amounts failed to rescue release (Figure S1A, lane 5). Interestingly, amounts of HA-Alix that prevented the rescue of HIV-1 PTAP- release also caused a significant decrease in wt HIV-1 release (Figure S1A, lane 9). Further analysis and quantification of this inhibition, shown in Figure 1, demonstrated that over-expression of increasing amounts of HA-Alix potently inhibited HIV-1 exit, as only ∼15% of virus was detected outside the cell (Figure 1B and 1C). Similar results were obtained when this experiment was performed with the HIV-1 strain BH10 (data not shown). Alix over-expression also decreased Gag processing as a modest accumulation of p25CA and other Gag intermediate processing products was seen (Figure 1B).

Next, we used transmission electron microscopy (TEM) to visually examine the defect in HIV-1 release caused by over-expression of Alix. HIV-1 was blocked at the plasma membrane where numerous arrested budding structures decorated the cell surface (Figure 1D). Over-expression of Alix arrested HIV-1 budding at various stages but most structures were arrested at early and mid-stages of budding (inset a). We also found aberrant and multi-budded particles (insets b and c) as well as some structures stopped in the late steps of budding (inset c). Similar results were observed with the HIV-1 BH10 strain (Figure S1B). These data suggested that over-expression of Alix led to interference with HIV-1 morphogenesis and budding. Interestingly, although PTAP/Tsg101 is considered to be the primary pathway utilized by HIV-1, over-expression of Alix efficiently decreased HIV-1 release suggesting that the inhibition is due to interference with the function and/or recruitment of a cellular factor common to both the PTAP/Tsg101 and LYPX_n_L/Alix budding pathways.

### Over-expression of an N-terminal fragment of Bro1 inhibits HIV-1 release

**Figure 2:**
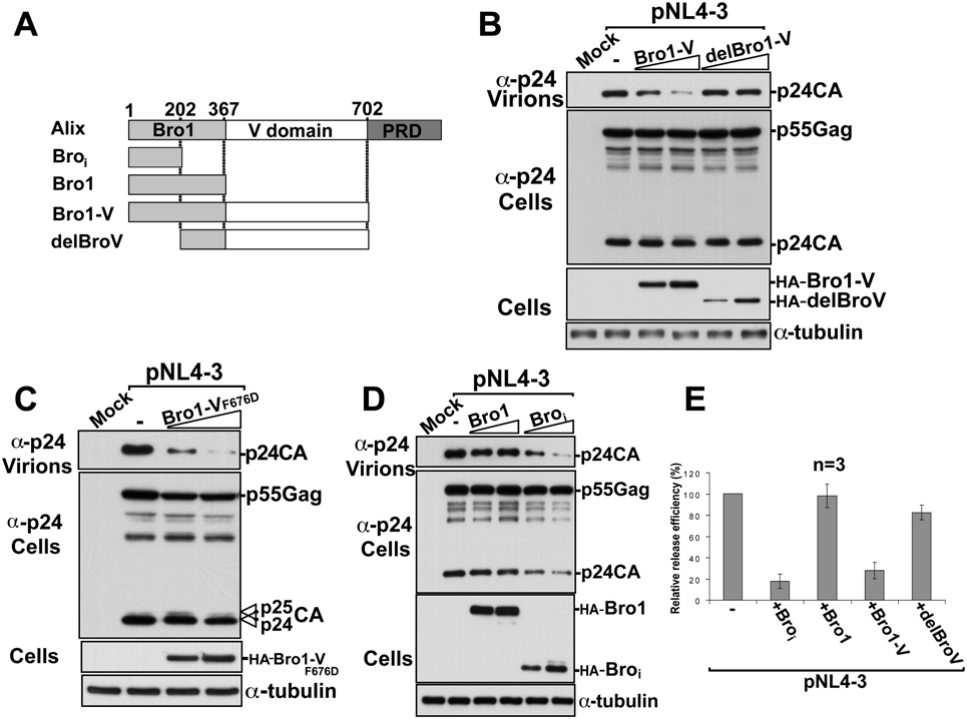
The N-terminal region of Bro1 inhibits the release of HIV-1. (A) Schematic representation of constructs used in the experiment. Numbers indicate positions of amino acid residues. (B, C, D) 293T cells were transfected with either pNL4-3 plasmid alone or with increasing amounts of the indicated dominant-negative fragments of Alix. Pelleted virions and cell lysates were analyzed by SDS-PAGE and western blot using the indicated antibodies. (B) Over-expression of Bro1-V but not delBroV inhibited virus-release. (C) The Bro1-V_F676D_ mutant retains inhibitory effect on HIV-1 release. (D) Bro_i_, but not Bro1, inhibits HIV-1 release. (E) Relative release efficiency of HIV-1 virions upon over-expression of each of the four N-terminal fragments of Alix. Error bars indicate the standard deviation from three separate experiments.

We tested fragments of the Alix protein to determine the role of the various domains in the inhibition the protein exerted on HIV-1 release. We first removed the C-terminal PRD to exclude the involvement of the numerous cellular partners known to bind this region, including Tsg101. We then focused on fragments containing the N-terminal Bro1 and the central V domain (Figure 2A) because these domains bind ESCRT-III and Gag, respectively, and were shown to play key roles in Alix function during virus release [44],[47],[60],[65]. Over-expression of Bro1-V efficiently inhibited HIV-1 release but had no detectable effect on Gag accumulation in the cell (Figure 2B). Removal of the N-terminal half of the Bro1 domain reversed the inhibitory effect of the Bro1-V fragment (Figure 2B), implying that binding via the V domain is not sufficient for inhibition in this context. To examine whether the V domain binding to Gag is involved in the Bro1-V block of HIV-1, we next tested the effect of the Bro1-V_F676D_ mutant that no longer binds the LYPX_n_L motif. We found that Bro1-V_F676D_ retained potent inhibition of HIV-1 release (Figure 2C), indicating that the Gag binding site in the V domain is dispensable for interference with viral exit in this context and suggesting that Bro1-V blocked HIV-1 production via regions located within the Bro1 domain. To map these inhibitory regions, we over-expressed the entire Bro1 domain and found that surprisingly, it had no inhibitory effect on HIV-1. In contrast, expression of a shorter fragment encompassing the first 202 residues of the Bro1 domain (Bro_i_) caused a potent inhibition of HIV-1 release (Figure 2D). Bro_i_ had little effect on Gag accumulation in the cell (Figure 2D). These findings identify the N-terminal 202 residues of the Bro1 domain as the region that drives inhibition of HIV-1 release (Figure 2E).

### Bro_i_ inhibits both the PTAP/Tsg101 and LYPX_n_L/Alix budding pathways

**Figure 3:**
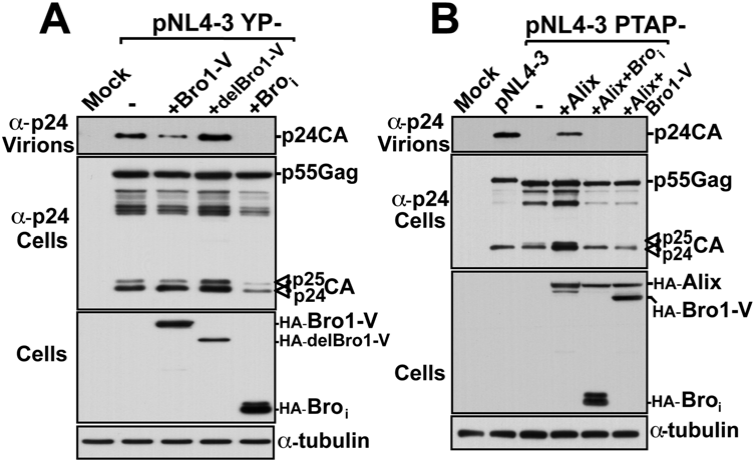
Bro_i_ and Bro1-V inhibit HIV-1 release mediated *via* both Tsg101 and Alix pathways. (A) Inhibition of an HIV-1 mutant lacking the LYPX_n_L motif. 293T cells were transfected with either pNL4-3 mutant lacking the LYPX_n_L motif (pNL4-3 YP-) alone or with the indicated dominant-negative fragment of Alix. Pelleted virions and cell lysates were analyzed by western blot using the indicated antibodies. (B) Interference with Alix driven rescue of an HIV-1 mutant lacking the PTAP motif. 293T cells were transfected with either wt pNL4-3 alone, with pNL4-3 mutant lacking the PTAP motif (pNL4-3 PTAP-) alone or with Alix in the presence or absence of the indicated dominant-negative fragment of Alix. Pelleted virions and cell lysates were analyzed by SDS-PAGE and western blot using the indicated antibodies.

To further examine the nature of the inhibition caused by the dominant negative fragments on HIV-1 release, we over-expressed Bro_i_ and Bro1-V with an HIV-1 lacking the LYPX_n_L motif (pNL4-3 YP-). In absence of the Alix binding site, this mutant HIV-1 relies only on the PTAP/Tsg101 pathway for viral exit. As with wild type pNL4-3, over-expression of delBroV had no effect on the release of NL4-3 YP- (Figure 3A). In contrast, over-expression of the Bro1-V inhibited NL4-3 YP- virus release and Bro_i_ exerted the most potent inhibition (Figure 3A). This indicated that over-expression of Bro_i_ (and Bro1-V) disrupted a key function in the PTAP/Tsg101 budding pathway.

To examine the effect of Bro_i_ and Bro1-V on HIV-1 release driven via the LYPX_n_L/Alix pathway, we used the budding defective HIV-1 PTAP- mutant. Over-expression of Alix has been shown to rescue the release of this mutant virus by acting through the LYPX_n_L motif [44],[47]. We reasoned that if the NC-Bro1 interaction is involved in the Alix-driven pathway as was recently suggested [60], Bro_i_ and Bro1-V might act as dominant negative fragments and interfere with the ability of Alix to rescue the PTAP- mutant. We tested this hypothesis by over-expressing Alix alone or with either fragment and found that in the presence of Bro_i_ or Bro1-V, Alix failed to rescue budding of the defective HIV-1 PTAP- mutant (Figure 3B, compare lanes 4, 5, and 6). Together these results indicate that Bro_i_ and Bro1-V exert a global inhibitory effect on HIV-1 budding and release.

### The NC domain of HIV-1 Gag is the primary target for Bro_i_ inhibition

**Figure 4:**
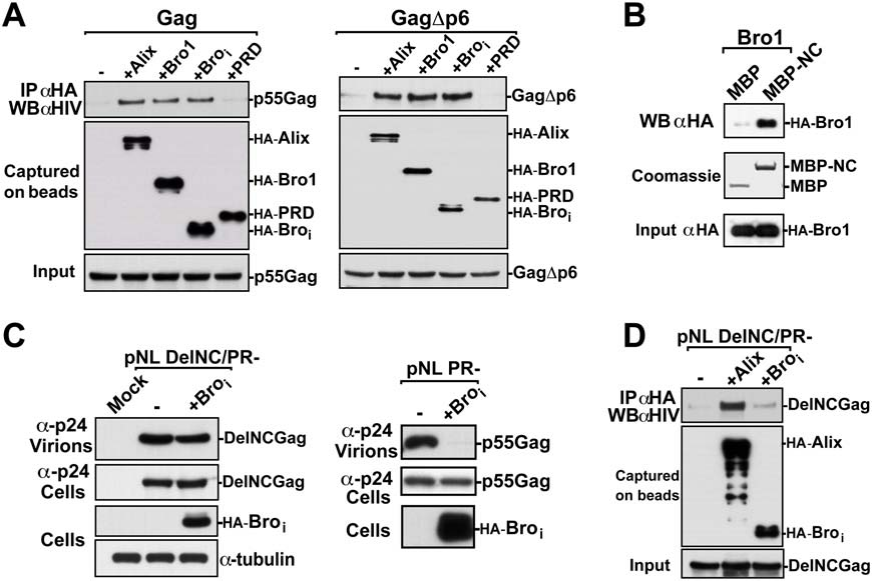
The NC domain of HIV-1 Gag is the primary target for Bro_i_ inhibition. (A) Bro_i_ and HIV-1 Gag co-immunoprecipitate in 293T cell lysates. 293T cells were co-transfected with either Gag-Pol (left panel) or GagΔp6-Pol (right panel) constructs along with expression vectors for HA-Alix, HA-Bro1, HA-Bro_i_, or HA-PRD. Cells were lysed in RIPA buffer and clear lysates were incubated with anti-HA antibody-conjugated beads. Both input and immunoprecipitated complexes were ran on SDS-PAGE for western blot analysis using the indicated antibodies. (B) The Bro1 domain of Alix binds NC *in vitro*. MBP and MBP-NC proteins were expressed in *E.coli*, immobilized on amylose resin and incubated with lysates from 293T cells expressing HA-Bro1. Captured Bro1 was detected with anti-HA antibody and MBP proteins were visualized by Coomassie blue staining. (C) The release of NL4-3 DelNC/PR- but not NL4-3 PR- is insensitive to the over-expression of Bro_i_. 293T cells were transfected with either pNL4-3 DelNC/PR- (Left panel) or pNL4-3 PR- (right panel) in presence or absence of HA-Bro_i_. Pelleted virions and cell lysates were analyzed by SDS-PAGE and western blot using the indicated antibodies. (D) Alix but not Bro_i_ co-immunoprecipitated with HIV-1 Gag DelNC. 293T cells were co-transfected with HIV-1 Gag DelNC/PR- along with either HA-Alix or HA-Bro_i_. Cells were lysed in RIPA buffer and clear lysates were incubated with anti-HA antibody-conjugated beads. Both input and immunoprecipitated complexes were ran on SDS-PAGE for western blot analysis using the indicated antibodies.

The results above indicated that Bro_i_ efficiently interfered with HIV-1 release, prompting the question as to whether Bro_i_ interacts directly with Gag. To examine this, Alix, Bro1, Bro_i_ and PRD were tested for their ability to interact with HIV-1 Gag (Figure 4A, left panel) and a GagΔp6 mutant (right panel) in immunoprecipitation assays. In contrast to PRD, Alix, Bro1 and Bro_i_ efficiently pulled-down Gag and the GagΔp6 mutant (Figure 4A). This demonstrated that in the cell, Bro1 binds HIV-1 Gag in a p6-independent manner and that the Bro_i_ region is sufficient for this interaction. Further examination by *in vitro* pull-down assays showed that NC binds the Bro1 domain of Alix (Figure 4B). These data confirmed that Alix contains a novel binding site for Gag located within Bro1, which is in agreement with the recent findings of Popov et al, 2008 [60]. Our results identify the first 202 residues of the Bro1 domain as sufficient for binding HIV-1 Gag.

Because the new Alix-Gag interaction is mediated via NC [60], we next examined whether the observed Bro_i_ interference with HIV-1 release occurs through the NC domain of Gag. For this, we assessed the effect of Bro_i_ on an HIV-1 mutant lacking the NC domain [62]. We used the HIV-1 DelNC/PR- mutant that recovered the ability to release viral particles because the protease (PR) was inactivated [62]. Over-expression of Bro_i_ had little effect on the release of HIV-1 DelNC/PR- mutant (Figure 4C, left panel), but retained a potent inhibitory effect on HIV-1 PR- that carries an intact NC domain (Figure 4C, right panel), implying that NC is the primary target for Bro_i_ inhibition. In support of this finding, Bro_i_ failed to immunoprecipitate the mutant DelNC Gag, which retained binding to full length Alix (Figure 4D). These results indicate that Bro_i_ inhibition of HIV-1 release is mediated *via* NC.

### Gag recruitment of Bro_i_ to the plasma membrane inhibits HIV-1 budding

**Figure 5:**
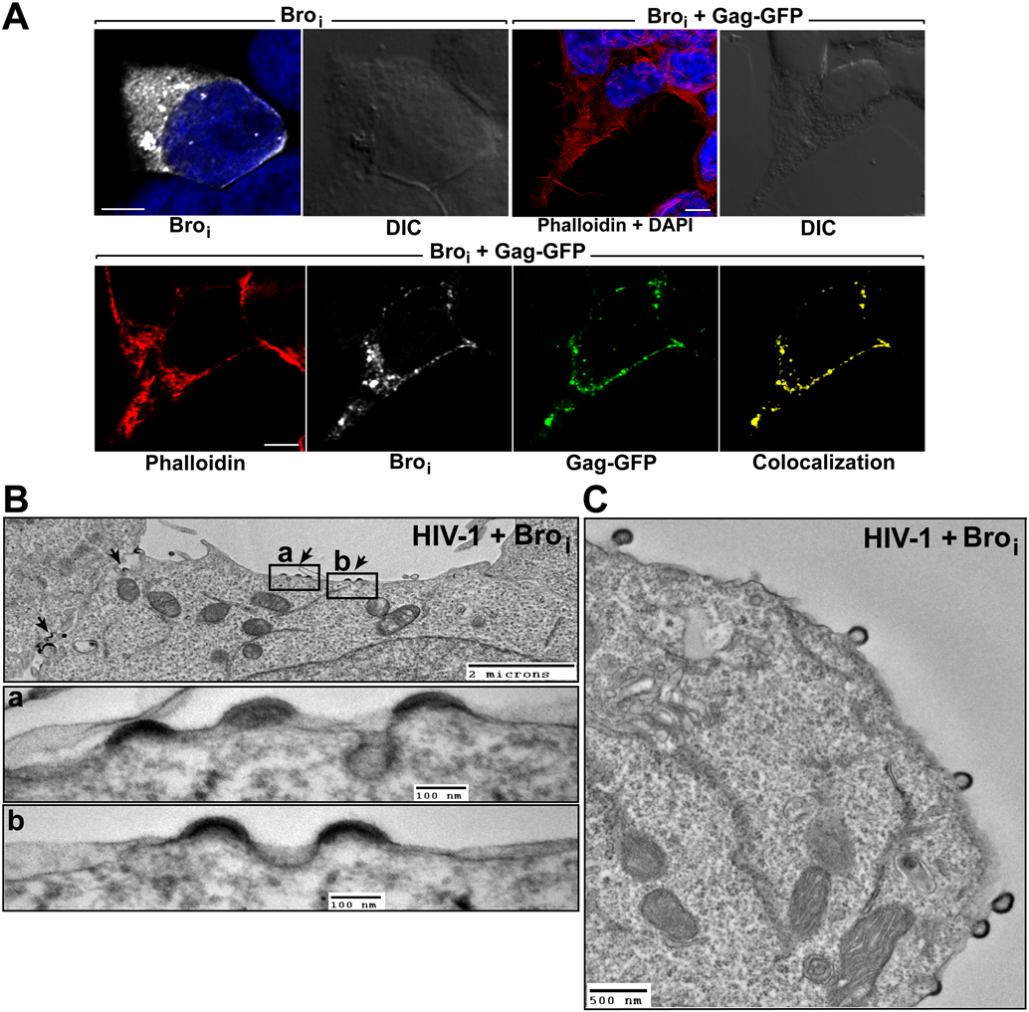
Bro_i_ is recruited by Gag to the plasma membrane and interferes with HIV-1 budding. (A) First two upper left panels show a cell expressing HA-Bro_i_ (white). The next panel shows a reconstructed 3D view of a stack of Z sections from a cell expressing HA-Bro_i_ and Gag-GFP. Lower panels show a single Z section of the same cell showing colocalization of Bro_i_ and Gag-GFP at the plasma membrane. Bro_i_ (white) was stained with a mouse monoclonal anti-HA antibody and an Alexa 633-conjugated anti-mouse antibody. Nuclei were counterstained with DAPI (blue). F-actin was stained with Alexa 568-conjugated phalloidin (red) to delineate cells. The colocalization channel (yellow) of Bro_i_ and Gag-GFP was built using Imaris software. Scale bar = 5 µm. (B, C) Electron micrographs of 293T cells co-transfected with pNL4-3 wt and HA-Bro_i_. (B) Arrested budding structures are indicated with black arrows. Two regions of interest in (a) and (b) show budding structures carrying electron-dense crescent-shaped material at a higher magnification. (C) HIV-1 budding structures tethered to the plasma membrane.

Since Bro_i_ interacts with Gag, we examined the cellular localization of Bro_i_ *vis-à-vis* HIV-1 Gag. Using confocal microscopy analysis, we found that Bro_i_ displayed a diffuse cytoplasmic distribution and localized to intracytoplamic vacuoles (Figure 5A). We next examined the localization of Bro_i_ vis-à-vis HIV-1 Gag in the cell and found that both proteins displayed a clear colocalization at the plasma membrane (Figure 5A, lower panels). This result indicated that HIV-1 Gag recruits Bro_i_ to the plasma membrane.

After establishing that HIV-1 Gag and Bro_i_ co-immunoprecipitated in the cell and colocalized at the plasma membrane, we sought to visualize the effect of Bro_i_ on HIV-1 morphogenesis and release from the cell using TEM. The over-expression of Bro_i_ led to the accumulation of multiple arrested budding structures at the plasma membrane (Figure 5B, a and b). We captured numerous crescent shaped particles, emerging outward to bud from the cytoplasm and aberrant, multi-budded structures that were tethered to the cell surface (Figure 5C and Figure S2A). The arrested budding particles appeared similar to those observed following the over-expression of a dominant negative version of Tsg101 [23] or Alix [65]. These findings demonstrate that Bro_i_ over-expression potently inhibits HIV-1 budding.

### Over-expression of Bro_i_ causes the formation of Class E compartments

**Figure 6:**
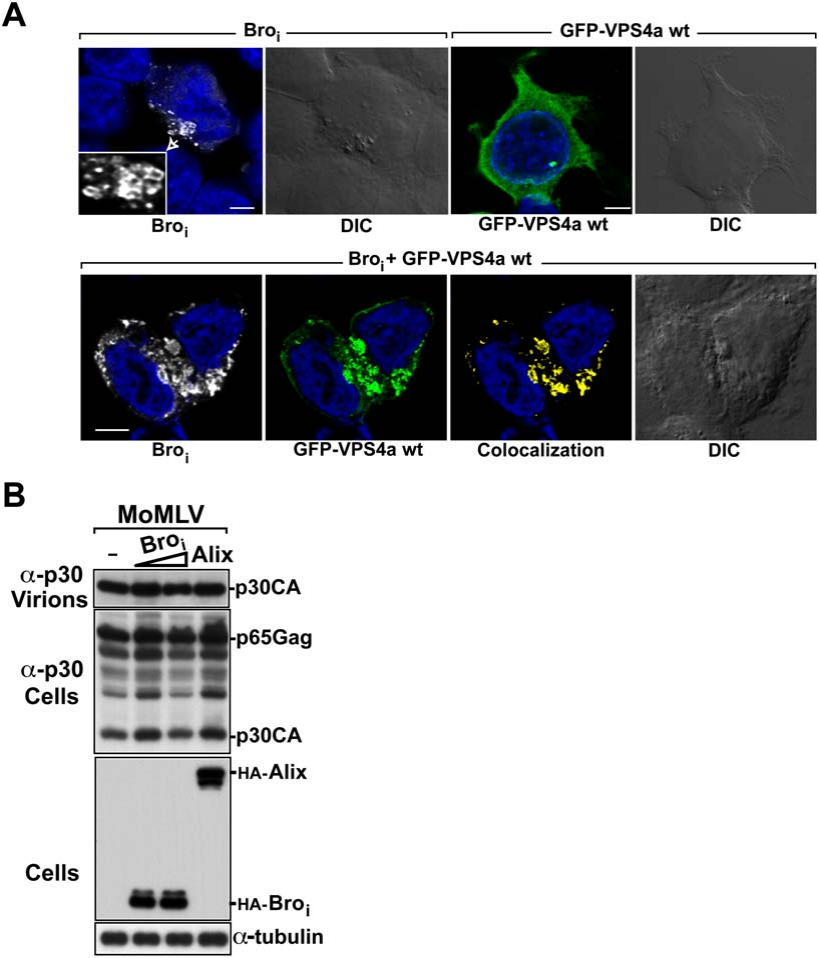
Over-expression of Bro_i_ causes the formation of Class E compartments but has no effect on MoMLV release. (A) The two upper left panels show a Z section of a cell expressing HA-Bro_i_ (white). Inset shows, at a higher magnification, an area of the cell (white rectangle) where HA-Bro_i_ associated with membranes of intracytoplasmic vacuoles. The two upper right panels show a Z section of a cell expressing GFP-VPS4a (green). Lower panels show Z sections of cells expressing both HA-Bro_i_ and GFP-VPS4a. Bro_i_ was stained with a mouse monoclonal anti-HA antibody and an Alexa 633-conjugated anti-mouse antibody. Nuclei were counterstained with DAPI (blue). The colocalization channel (yellow) of Bro_i_ and GFP-VPS4a was built using Imaris software. Scale bar = 5 µm. (B) Over-expression of Bro_i_ does not inhibit the release of MoMLV. 293T cells were transfected with either pNCA plasmid alone or with Bro_i_ and Alix. Pelleted virions and cell lysates were analyzed by western blot using the indicated antibodies.

Bro_i_ over-expression exerted a potent inhibition on HIV-1 budding *via* the NC domain in Gag. This suggested that Bro_i_ binding interfered with p6 recruitment of one or multiple members of the ESCRT machinery that function in HIV-1 budding and release. Since HIV-1 release and MVB inward budding both require the recruitment of members of the same machinery for membrane fission, we asked whether Bro_i_ is recruited to endosomal compartments and interferes with MVB vesicle formation. Bro_i_ displayed a diffuse cytoplasmic staining (Figure 5 and Figure 6). In addition, Bro_i_ also accumulated in large intracytoplamic vacuoles reminiscent of enlarged endosomal vacuoles (Figure 6A). Interestingly, Bro_i_ associated with the membrane of these vacuoles (Figure 6A, inset). To check whether these intracytoplasmic vacuoles were aberrant Class E compartments, we examined Bro_i_ localization in presence of the AAA-type ATPase VPS4a, which is a key player in MVB function. Bro_i_ affected the intracellular distribution of VPS 4a protein and caused it to accumulate in the enlarged endosomal compartments (Figure 6A, lower panels). This result indicates that Bro_i_ over-expression caused the formation of class E compartments. To examine whether Bro_i_-mediated inhibition is specific to HIV-1 and not due to a general toxicity that adversely affected the cell, we tested the impact of Bro_i_ and Alix over-expression on the release of the Moloney Murine Leukemia Virus (MoMLV), a gammaretrovirus that requires ESCRT proteins but not Tsg101 for viral egress [16]. Expression of increasing amounts of Bro_i_ (comparable to those used in Figure 2) or Alix had no effect on the release of MoMLV (Figure 6B), demonstrating that the effect of Bro_i_ on HIV-1 release is specific.

The Bro1 domain of Alix binds CHMP4 isoforms, the ESCRT-III components that have been shown to be essential for HIV-1 budding and release [44],[47]. We hypothesized that Bro_i_ might have interfered with CHMP4 recruitment to Gag by titration of the available pool of CHMP4 proteins in the cytoplasm. To test this hypothesis, we examined the ability of Bro_i_ to bind the three isoforms of CHMP4 using immunoprecipitation assays. Although Bro_i_ carries part of the CHMP4 binding interface [44],[46],[47],[66], it was unable to interact with the three CHMP4 isoforms (Figure S3A, S3B, and S3C), even though it retained binding to MAP/RabGAPLP, a known cellular partner of the Bro1 domain of Alix [67] (Figure S3D). The inability of Bro_i_ to bind CHMP4 isoforms was also observed in yeast two hybrid assays (data not shown). We conclude that Bro_i_ interference with HIV-1 budding is not due to the titration of CHMP4 proteins.

### Over-expression of Bro1-V inhibits HIV-1 budding in a CHMP4–dependent manner

**Figure 7:**
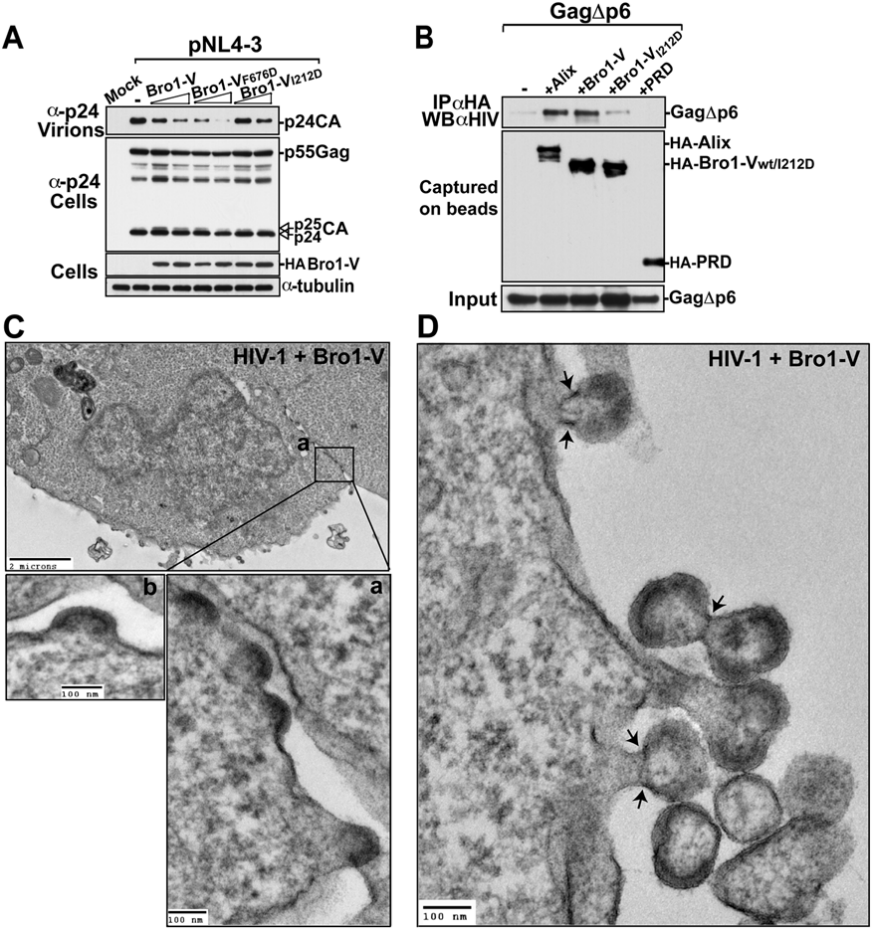
Over-expression of Bro1-V inhibits HIV-1 budding in a CHMP4–dependent manner. (A) The inhibitory effect of Bro1-V is dependent on its CHMP4 binding site. 293T cells were transfected with either pNL4-3 plasmid alone or with increasing amounts of the indicated mutants of Bro1-V. Pelleted virions and cell lysates were analyzed by SDS-PAGE and western blot using the indicated antibodies. (B) Bro1-V but not Bro1-V_I212D_ co-immunoprecipitates with HIV-1 GagΔp6. 293T cells were co-transfected with GagΔp6-Pol along with expression vectors for HA-Alix, HA-Bro1-V, HA-Bro1-V_I212D_ and HA-PRD. Cells were lysed in RIPA buffer and clear lysates were incubated with anti-HA antibody-conjugated beads. Both input and immunoprecipitated complexes were ran on SDS-PAGE for western blot analysis using the indicated antibodies. (C–D) Electron micrographs of 293T cells co-transfected with pNL4-3 wt and HA-Bro1-V. (C) The black boxes indicate two regions of interest shown in (a) and (b) at a greater magnification. (D) HIV-1 arrested budding structures. Arrows indicate the electron-dense “ring-like” structure visible at the budding neck of arrested particles.

Over-expression of Bro1-V caused inhibition of HIV-1 release. Moreover, mutation of the Gag binding site in Bro1-V (Bro1-V_F676D_) had no effect on the ability of this fragment to inhibit HIV-1 release, confirming that Bro1-V interference with HIV-1 release is primarily mediated via the Bro1 domain. To address whether this inhibition is related to Bro1 recruitment of CHMP4, we constructed Bro1-V_I212D_, a mutant that has lost the ability to bind CHMP4 isoforms, and tested its effect on HIV-1 release. Bro1-V_I212D_ mutant had little to no effect on HIV-1 release (Figure 7A) indicating that Bro1-V inhibition of HIV-1 production required an intact CHMP4 binding site in Bro1. Surprisingly, whereas Bro1-V strongly bound Gag missing the p6 domain, Bro1-V_I212D_ lost the ability to bind this truncated Gag (Figure 7B), while retaining binding to full length Gag (data not shown). These results demonstrated that the mutation of the CHMP4 binding site in Bro1 affected its ability to bind Gag via NC but not p6 and that the Bro1-V inhibition of HIV-1 release is due to the binding of Bro1 to NC and requires an intact CHMP4 binding site.

To visualize the Bro1-V effect on virus release, we examined HIV-1 release in cells expressing Bro1-V by TEM. This analysis showed multiple cells carrying a high number of arrested HIV-1 budding structures that decorated the cell surface. Because the majority of the arrested particles displayed an early budding phenotype, viral morphogenesis was examined at time 32 and 48 hours post-transfection to evaluate the inhibition caused by Bro1-V overtime. At both time points, over-expression of Bro1-V impaired HIV-1 budding, allowing the capture of nascent particles carrying crescent shaped electron-dense material (Figure 7C). Arrested budding structures that remained attached to the plasma membrane and displayed an aberrant morphology were also observed. Remarkably, at 48 h, some of these structures showed a “ring-like” structure or a filament surrounding the budding neck of the particles (Figure 7D and Figure S2B), that is reminiscent of those caused by dominant negative variants of ESCRT proteins [21]. Together these data indicate that Bro1-V interferes with HIV-1 budding, and that this inhibition requires binding to NC and an intact CHMP4 binding site.

### The Bro1 domain of Alix links Gag to ESCRT-III members via NC

**Figure 8:**
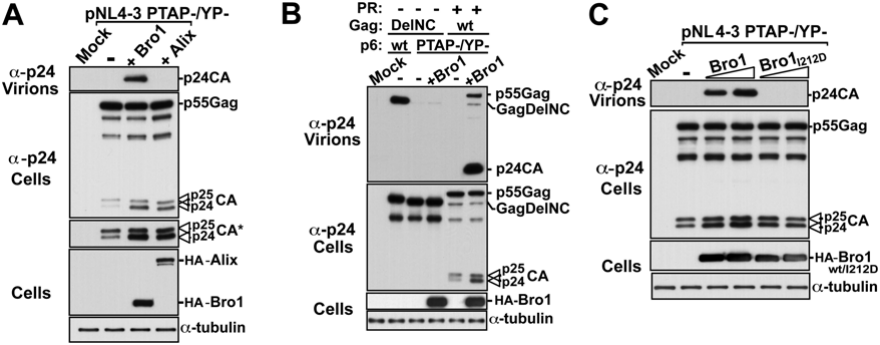
The Bro1 domain of Alix links Gag to ESCRT-III *via* NC. (A) The isolated Bro1 domain, but not the full-length Alix, rescues the release of the NL4-3 PTAP-/YP- virus. 293T cells were transfected with either pNL4-3 PTAP-/YP- plasmid alone or with HA-Bro1 or HA-Alix. Pelleted virions and cell lysates were analyzed by SDS-PAGE and western blot using the indicated antibodies. The asterisk indicates a longer exposure showing details of p24/p25 processing in the cells. (B) The rescue of the NL4-3 PTAP-/YP- double mutant virus depends on an intact NC domain in Gag. HA-Bro1 was co-expressed in 293T cells with either pNL4-3 DelNC PTAP-/YP-/PR-, which lacks NC (DelNC) and carries an inactive viral protease (PR-) (lane 4) or pNL4-3 PTAP-/YP- (lane 6) as a control. Pelleted virions and cell lysates were analyzed by SDS-PAGE and western blot using the indicated antibodies. (C) The rescue of NL4-3 PTAP-/YP- release by Bro1 requires an intact CHMP4 binding site. 293T cells were transfected with either pNL4-3 PTAP-/YP- plasmid alone or with increasing amounts of HA-Bro1 (lanes 3–4) or HA-Bro1_I212D_ mutant (lanes 5–6). Pelleted virions and cell lysates were analyzed by western blot using the indicated antibodies.

Our studies demonstrated that Gag recruited Alix via its Bro1 domain in a p6 independent manner (Figure 4). In addition, we found that over-expression of Bro1 caused no inhibition of HIV-1 (Figure 2). In fact, expression of higher amounts of Bro1 stimulated HIV-1 release (See Figure S5D). This suggested that Bro1 binding to Gag might be sufficient to mediate L domain function. Consistent with this hypothesis we found that, like Alix, over-expression of the isolated Bro1 domain rescued the release of the HIV-1 PTAP- defective mutant (data not shown). This rescue was independent of the presence of the LYPX_n_L motif in p6 since Bro1 over-expression rescued the release of an HIV-1 mutant lacking both PTAP and LYPX_n_L motifs (NL4-3 PTAP-/YP-) (Figure 8A). Bro1 over-expression also corrected the typical processing defect associated with L domain impairment, as seen by further maturation of p25CA into p24CA product (Figure 8A, see darker exposure of this panel). In contrast, Alix rescue of the L domain defective double mutant was nearly undetectable (Figure 8A).

**Figure 9:**
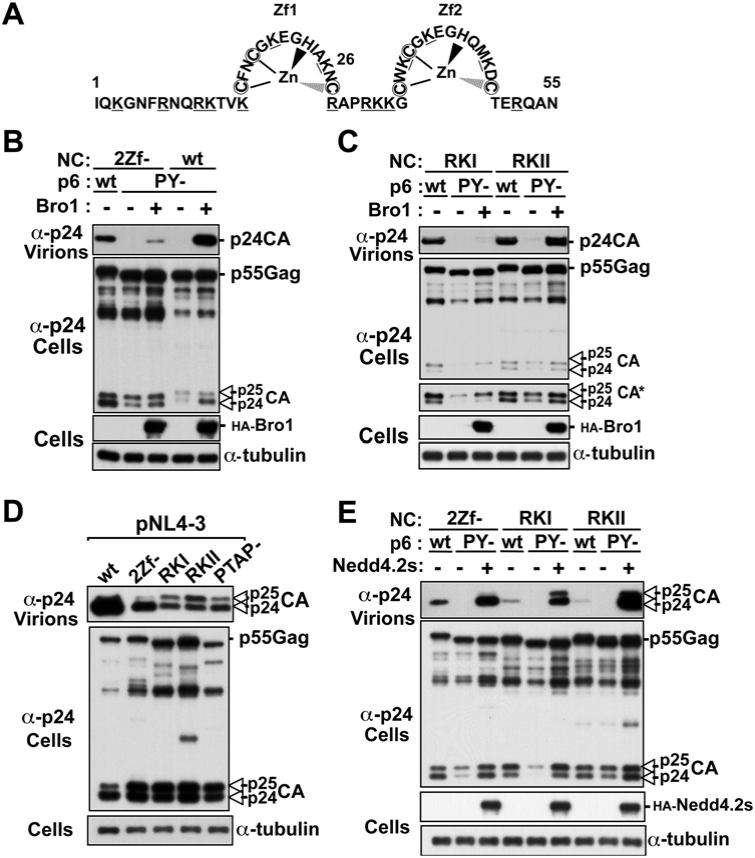
Mapping of regions in NC important for HIV-1 release. (A) Schematic representation of NC residues (1–55) with the two zinc fingers (Zf). Cysteine residues (C) changed to serine in the 2Zf- mutant are circled whereas arginine and lysine residues (R, K) were substituted to alanine in RKI (K3 to K26) and RKII (K26 to K52) mutants are underlined. (B, C) Zinc fingers and basic residues in the N-terminal portion of NC are necessary for the rescue of HIV-1 PTAP-/YP- (here named PY-) release by Bro1. (B) 293T cells were transfected with either pNL4-3 2Zf-, which carries disrupted zinc fingers (lane 1), or pNL4-3 2Zf-/PY- (lanes 2–3). pNL4-3 PY- is used (lanes 4–5) as a control for Bro1 rescue. In lane 3, co-transfection with pNL4-3 2Zf-/PY- and HA-Bro1 partially rescues viral release. (C) 293T cells were transfected with either pNL4-3 RKI (lanes 1-3) or pNL4-3 RKII (lanes 4–6) carrying either wt p6 (lanes 1 and 4) or a (PY-) p6 (lanes 2–3 and 5–6) and HA-Bro1 to examine the ability of Bro1 to rescue viral release. The asterisk indicates a longer exposure showing p24/p25 doublets in cell extracts. (D, E) Release defect of 2Zf-, RKI, RKII mutants is rescued by over-expression of Nedd4.2s. (D) 293T cells were transfected either with pNL4-3 (lane 1) or 2Zf-, RKI, RKII, and PTAP- mutant constructs (lanes 2–5). (E) 293T cells were transfected with either 2Zf-, RKI, RKII (lanes 1, 4 and 7) or their (PY-) mutant counterparts alone (lanes 2, 5, and 8) or with Nedd4.2s (lanes 3, 6, and 9). Pelleted virions and cell lysates were analyzed by SDS-PAGE and western blot using the indicated antibodies.

To examine whether the rescue of the HIV-1 double mutant by Bro1 was mediated via the NC domain in Gag, we performed the Bro1 rescue assay with either NL4-3 PTAP-/YP- or with NL4-3 PTAP-/YP- lacking NC (DelNC) and carrying an inactive protease (PR-). We first showed that alterations of PTAP and LYPX_n_L motifs in the DelNC/PR- virus ablated release (Figure 8B, lane 3), demonstrating that this virus requires L domains to exit the cell. Over-expression of Bro1 failed to rescue the NL4-3 PTAP-/YP- DelNC/PR- mutant (Figure 8B) indicating that Bro1-mediated rescue required an intact NC domain in Gag. To verify whether Bro1 binding to NC recruited CHMP4 proteins, we next tested Bro1_I212D_, a Bro1 mutant carrying a disrupted CHMP4 binding site, for its ability to rescue the HIV-1 PTAP-/YP- double mutant. Bro1_I212D_ failed to rescue the release of the defective HIV-1 double mutant (Figure 8C) although this fragment retained binding to Gag and was found incorporated in VLPs (Figure 9B and data not shown). These results demonstrate that Bro1 binding to NC links Gag directly to the CHMP4 proteins, which are required for HIV-1 release. The data also show that Bro1 alone can facilitate HIV-1 release in the absence of L domain motifs PTAP and LYPX_n_L, indicating that the Bro1 domain is the smallest functional unit of Alix.

We found that Alix rescue of the double mutant HIV-1 PTAP-/YP- was dramatically weaker in comparison to Bro1 rescue (Figure 8A). Therefore, in the context of full length Alix, binding to Gag via both the Bro1 and V domains is required for its function in HIV-1 release. The finding that Bro1 bound NC directly (Figure 4B) and rescued release of the double mutant HIV-1 PTAP-/YP- virus in an NC-dependent manner (Figure 8A and 8B) is strong evidence for a role of an NC-Bro1 interaction in HIV-1 budding. Bro1 rescue of the double mutant HIV-1 PTAP-/YP- via NC requires an intact CHMP4 binding site (Figure 8C) demonstrating that NC is the site through which a Bro1-CHMP4 complex is recruited by HIV-1 Gag to release virus via the LYPX_n_L/Alix pathway.

### The Bro1 domain of Alix specifically rescues the release of HIV-1 PTAP-/YP- mutant

In the cell, other proteins have been described to carry defined Bro1 domains. To test whether other Bro1-related proteins function in HIV-1 release in a manner similar to Alix, we selected HD-PTP, Rhophilin-2 and Brox, three cellular proteins that share structural similarities with Alix [46],[68],[69],[70]. Although these proteins carry defined Bro1 domains, only the Bro1 domains of HD-PTP and Brox bind CHMP4 proteins. In contrast to Alix, over-expression of HD-PTP, Brox and Rhophilin-2 failed to rescue the release of the HIV-1 PTAP- mutant (data not shown).

Since the isolated Bro1 domain of Alix was able to efficiently rescue HIV-1 release in a p6-independent manner and stimulate HIV-1 release (Figure 8 and Figure S5D), we isolated the Bro1 domains of HD-PTP and Rhophilin-2 (Figure S4) and examined their ability to function in HIV-1 release. We first tested whether the Bro1 domains of HD-PTP and Rhophilin-2, named HDBro1and RhoBro1 hereafter (Figure S5A), bind Gag. Using immunoprecipitation assays (Figure S5B), we found that both domains captured Gag in mammalian cell extracts. We next examined whether this binding alone is sufficient to link Gag to the downstream ESCRT-III machinery and facilitate HIV-1 release. Over-expression of HDBro1, RhoBro1 or Brox (data not shown) did not rescue the release of HIV-1 PTAP-/YP- but actually inhibited Gag processing as little to no p24CA protein was detected in cell extracts (Figure S5C). These results demonstrate that although the NC domain of Gag recognizes other cellular Bro1 domains, only the Bro1 domain of Alix specifically functions in HIV-1 release. The finding that HDBro1 and Brox (data not shown), which also bind CHMP4b proteins [68],[71], failed to rescue the HIV-1 PTAP-/YP- mutant, indicates that the Bro1-CHMP4 complex recruited via NC operates in a context-dependent manner. This result suggests that in addition to the CHMP4 binding site, other regions within the Bro1 domain of Alix are necessary for its function in the release of HIV-1 virions from the cell. Collectively, these data indicate that the rescue of HIV-1 budding defects by the Bro1 domain of Alix is efficient and specific.

### Mapping of NC regions required for Bro1 function in HIV-1 release

Recent studies demonstrate that disruption of both zinc fingers in NC inhibits Alix interaction with Gag and facilitation of virus release [60]. To map regions in NC involved in the recruitment of the Bro1 domain of Alix, we used mutations in NC that disrupt both zinc fingers (Figure 9A). In this experiment, the ability of the Bro1 domain of Alix to rescue wild type or the zinc finger mutant (2Zf-) [72],[73] lacking both PTAP and LYPX_n_L L domains was examined. In contrast to the HIV-1 L domain double mutant (PY-) carrying a wild type NC, the 2Zf- triple mutant was only weakly rescued by Bro1 over-expression (Figure 9B), demonstrating that NC zinc fingers play an important role in Bro1-mediated rescue of HIV-1 release. However, these mutations reduced but did not completely prevent Bro1 from rescuing viral release, suggesting that other regions in NC may be involved in Bro1 recruitment.

To search for additional regions in NC involved in Bro1 recruitment and function in HIV-1 release, we generated HIV-1 constructs carrying mutations of basic residues scattered throughout NC, because alterations of these residues were previously shown to affect virus release [61],[63],[64]. Substitution of seven lysine and arginine residues in the first (mutant RKI) or the second half of NC (mutant RKII) to alanines (Figure 9A), combined with alterations of the PTAP and LYPX_n_L motifs led to a complete loss of viral release (Figure 9C). In contrast to RKI mutant however, RKII virus release was rescued with the over-expression of Bro1 (Figure 9C) demonstrating that basic residues in the N-terminal half of NC are required for Bro1 facilitation of HIV-1 release and suggesting that this region of NC is involved in the recruitment of Bro1.

Mutations of basic residues throughout NC (mutants RKI and RKII) or in zinc fingers caused a severe decrease in the release of virions carrying an intact PTAP motif (Figure 9D). For this experiment, the level of virus release, as well as Gag processing in the cell, was examined by running protein extracts in conditions that allow good resolution of the p25/24 CA doublet. The pattern obtained with NC mutants RKI and RKII was strikingly similar to that of the PTAP motif mutant virus (PTAP-) (Figure 9D, lanes 3–5). This result indicates that in the context of NC mutants, the PTAP motif is not sufficient to drive virus release, and suggests that NC and the PTAP motif play cooperative roles in viral release. The effect of mutations in NC on virus production was previously attributed to assembly and/or virus budding defects [61],[74]. We hypothesized that NC mutations might interfere with virus budding by preventing the recruitment of cellular components involved in PTAP-mediated HIV-1 release. If this is the case, we reasoned that release defects caused by mutations in NC should be alleviated by recruitment, via regions in Gag outside of the NC domain, of cellular components known to facilitate viral release. In support of this hypothesis, we found that over-expression of Nedd4.2s, an isoform of the E3 ubiquitin ligase Nedd4.2 that was recently shown to stimulate the release of an HIV-1 variant missing L domains and NC [75],[76], efficiently rescued the release of 2Zf-, RKI, and RKII mutants lacking the PTAP and LYPX_n_L motifs (Figure 9E). This result demonstrates that NC plays a key role in connecting HIV-1 Gag to components of the cellular budding machinery necessary for HIV-1 release.

## Discussion

In this study, we find that the over-expression of Alix causes a severe reduction of HIV-1 release. Alix-mediated inhibition is independent of the Gag and Tsg101 binding sites, located within the V and the PRD domains respectively, suggesting that the Bro1 domain is responsible for this inhibition. These results reveal that Bro1 contains a novel binding site for Gag, which is in agreement with data reported by Gottlinger and colleagues [60]. We extend these observations with multiple findings that support a role for the Bro1 domain in HIV-1 budding and release. Specifically, we find that the over-expression of Bro1 rescues the release of an HIV-1 mutant lacking both the PTAP and LYPX_n_L motifs, and that this rescue provides the first direct evidence that Gag contains a site outside of p6 that mediates virus budding. Importantly, Bro1-driven rescue of HIV-1 release requires the CHMP4 binding site in Bro1 and an intact NC domain in Gag, demonstrating that NC is a novel site that links Gag to the cellular machinery required for HIV-1 release. A dominant negative version of Bro1, Bro_i_, potently inhibits HIV-1 release driven via either the PTAP/Tsg101 or the LYPX_n_L/Alix pathway. Bro_i_ mediated inhibition is NC-dependent and arrests HIV-1 budding at the plasma membrane. Interestingly, mutations in NC that prevent the Bro1-mediated rescue of an HIV-1 lacking both p6-L domains also decrease HIV-1 particle production. Moreover, release defects caused by mutations in NC are corrected by linking HIV-1 to the host budding apparatus via over-expression of Nedd4.2s, a cellular protein that was recently described to link Gag to the ESCRT machinery via interactions outside of the NC and p6 domains. Our studies indicate that NC plays a pivotal role in p6-mediated Late domain functions by controlling the recruitment of the host budding machinery that is necessary for HIV-1 egress.

### The role of NC in the LYPX_n_L/Alix budding pathway

A role for NC in HIV-1 budding driven via the LYPX_n_L/Alix pathway has been recently reported [60]. This new function requires residues in NC believed to interact with the Bro1 domain of Alix. Consistent with this notion, Bro1 is sufficient to rescue the release of an HIV-1 mutant lacking only the PTAP motif or both PTAP and LYPX_n_L motifs. Because this rescue requires a CHMP4 binding site in Bro1, the results clearly show that Bro1 links Gag to members of ESCRT-III *via* an interaction with NC, thus identifying Bro1 as the smallest functional unit of Alix. These findings show that, in addition to its known Gag binding site in the V domain, Alix contains a second binding site for Gag located within Bro1 that mediates the recruitment of CHMP4 proteins necessary for HIV-1 separation from the plasma membrane.

In contrast to Bro1, Alix does not efficiently rescue the defective HIV-1 double mutant PTAP-/YP- even though it retains binding to the Gag mutant lacking p6 through the Bro1 domain. Therefore, when expressed in the context of Alix, the Bro1 domain is not sufficient, or not available, to function when the V domain is not bound to the LYPX_n_L motif, possibly because the V and PRD domains may be masking the functional site within Bro1. Since removal of the V and PRD domains generates a functional Bro1 domain, it is conceivable that the binding of the V domain of Alix to p6 results in conformational changes that reveal functional sites (i.e. CHMP4 binding site) within the Bro1 domain. The functional crosstalk between the Bro1 and V domains is also apparent in the binding properties of the Bro1-V fragment to the p6-deleted Gag. Bro1-V binds Gag in absence of p6 indicating that the interaction is mediated primarily via Bro1. Disruption of the CHMP4 binding site in Bro1-V negatively affects this interaction, although the isolated Bro1 domain carrying the same disruption (Bro1_I212D_) retains binding to Gag. This suggests that the presence of the V domain weakens Bro1 binding to NC. Therefore, the Bro1 domain interaction with NC is highly influenced by the adjacent V domain, with which it has been shown to form a tight structural unit [44]. Collectively, these observations emphasize the functional and structural interconnection between the Bro1 and the V domains, which bind NC and p6, respectively.

**Figure 10:**
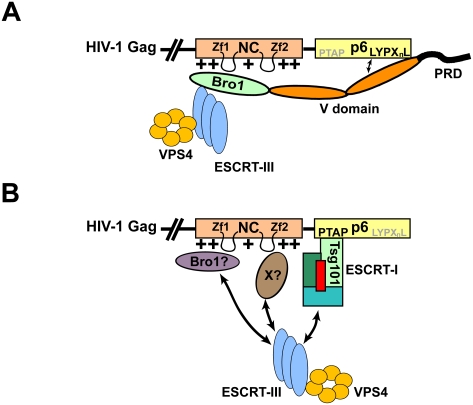
A model for NC cooperation with the PTAP/Tsg101 and LYPX_n_L/Alix budding pathways. (A) Role of NC in the LYPX_n_L/Alix pathway: NC interacts with the Bro1 domain of Alix through its N-terminal basic residues and zinc fingers to recruit the essential downstream budding machinery components, ESCRT-III and VPS4, to promote virus egress. (B) Role of NC in the PTAP/Tsg101 pathway: basic residues throughout NC as well as zinc fingers may be required for HIV-1 budding via the PTAP motif, because they participate in the recruitment of members of the cellular budding machinery that include a Bro1-containing protein (Bro1? or Alix) and possible additional host factor(s) (X?) that cooperate with PTAP-bound complexes to facilitate virus release. (+) symbols represent basic residues in NC, (Zf): zinc finger.

Our studies indicate that the zinc fingers within NC participate in Bro1 interactions as their disruption decreases the ability of Bro1 to rescue the release of HIV-1 lacking both L domain motifs (Figure 9). However, since Bro1 retained some level of activity in this assay, other regions in NC appear to be involved in Bro1-NC interactions. In fact, our data identify the N-terminal portion of NC as the functional determinant for Bro1-NC interactions, since mutations of basic residues in this region prevented Bro1, and Alix (data not shown), rescue of budding defects. Interestingly, the zinc finger located within the N-terminal portion of NC is also known to play a key role in the Bro1 domain recruitment and Alix facilitation of HIV-1 release [60], suggesting that both the zinc finger and basic residues in this region of NC participate in the recruitment of the Bro1 domain of Alix. These findings identify the N-terminal portion of NC as necessary for the recruitment of Bro1, an essential facilitator of HIV-1 release via the LYPX_n_L/Alix pathway (Figure 10A).

### The role of NC in the PTAP/Tsg101 budding pathway

In the current model for HIV-1 budding, the p6-located L domain motif PTAP is considered the primary recruitment site for cellular factors implicated in HIV-1 budding and release. In this study, we present evidence supporting the notion that NC cooperates with the PTAP motif to facilitate HIV-1 release. Interestingly, other studies show that PTAP function within HIV-1 Gag or within heterologous retroviral Gag, depends on its location within the polyprotein [58],[59], suggesting that PTAP might require other regions in Gag to facilitate viral budding and release. When transplanted into the retroviral Gag of RSV [58], MLV [59]or EIAV [77], the PTAP sequence of HIV-1 p6 retains some level of release-driving activity. Notably, in all these Gag chimeras the PTAP motif was either transplanted as part of a sequence that also carries the HIV-1 NC domain [59], or was placed next to the NC domain of the “host” Gag [58],[59],[77]. In contrast, when expressed next to the MA domain within RSV or MoMLV Gag, the PTAP motif of HIV-1 rescues only ∼30% of virus release [58],[59]. Additionally, replacement of NC with a leucine zipper causes a severe reduction in virus release [61],[62],[64],[78]. In fact deletion or mutations in NC cause a severe inhibition of virus release (Figure 9 and [61],[62],[63],[64]), although NC-mutated Gag proteins carry intact L domains and retain the ability to assemble particles. Interestingly, both NC and the PTAP motif are required for efficient budding in the context of a minimal Gag construct [20]. Thus, within HIV-1 Gag neither NC nor the PTAP motif is individually sufficient to mediate virus exit [10],[11],[61],[62],[63],[64], suggesting that their functions are inter-dependent.

As evidence for NC-PTAP cooperation in HIV-1 release, we find that Bro_i_ and Bro1-V, two fragments of Alix that exhibit dominant negative properties, potently inhibit the release of a PTAP-dependent HIV-1. We confirmed that Bro_i_ is responsible for the inhibition because removal of the first 202 N-terminal residues (corresponding to Bro_i_) of Bro1-V reverses the fragment's inhibitory effect on HIV-1 release. We show evidence that the effect of Bro_i_ is specific to HIV-1 and not due to an indirect or toxic effect on the cell, because it has no inhibitory effect on the release of MoMLV. Additionally, deletion of the NC domain confers resistance to Bro_i_ inhibition, demonstrating that the NC domain of Gag is the site targeted by Bro_i_. We therefore interpret the Bro_i_ effect on viral release as resulting from an interference with a NC function necessary for HIV-1 budding driven via the PTAP motif. In fact, over-expression of Bro_i_ or Bro1-V interferes with HIV-1 budding and release from the plasma membrane as numerous arrested budding structures decorate the cell surface. In addition, Bro1-V causes the appearance of arrested budding structures that carry an electron-dense filament surrounding their budding neck, a phenotype that was previously seen following interference with the function of ESCRT/MVB proteins [19],[20],[21],[51],[52],[53]. Interestingly, Bro_i_ over-expression causes the formation of Class E compartments. Similarly, RNAi-depletion of Tsg101 also leads to the formation of aberrant endosomal compartments [79] and inhibition of HIV-1 release, but not MoMLV [16]. Because Bro_i_ has no effect on MoMLV release, we propose that Bro_i_ inhibition results from an interference with the function/recruitment of components of the cellular budding machinery that specifically cooperates with the PTAP/Tsg101 budding pathway in HIV-1 egress. The finding that Bro_i_ is found associated with endosomal membranes, even though it cannot bind CHMP4 proteins suggests that this portion of Alix associates with a cellular factor or factors that function in endosomal compartments.

It is therefore possible that alteration of NC causes an effect similar to that exerted by Bro_i_ (or Bro1-V) because both render NC unavailable to recruit a host factor(s) that cooperates with PTAP in order to facilitate virus release. In fact, we find that mutations of basic residues in the NC domain of Gag cause a severe decrease in HIV-1 release. Remarkably, the release of these NC-mutated viruses is rescued by Nedd4.2s, a cellular protein that stimulates HIV-1 release in the absence of NC and L domain motifs PTAP and LYPX_n_L [75],[76]. These data suggest that mutations in NC inhibit budding by disabling the recruitment of cellular proteins necessary for virus egress, a defect that can be corrected by linking Gag to ESCRT proteins via a parallel pathway (i.e Nedd.4.2s association with Gag). Interestingly, mutations of basic residues in the first half of NC (mutant RKI) also prevent Bro1-mediated rescue of HIV-1 release, demonstrating that this NC-mutant virus lost the ability to interact with Bro1. These data suggest that NC may recruit the Bro1 domain of Alix to cooperate with the PTAP motif and facilitate release. However, Alix has been reported to be dispensable for PTAP function in 293T cells because PTAP retains activity when Alix is RNAi-depleted from the cell [43]. These observations raise the possibility that in 293T cells, HIV-1 may compensate for the lack of Alix by recruiting other cellular Bro1 domain-containing proteins.

The role of NC in HIV-1 release appears to be quite complicated, and is not limited to the recruitment of Alix, since the release defect caused by mutations of basic residues in the C-terminal half of NC (mutant RKII) (this study, [61],[63],[64]), can be rescued by Bro1, as well as Nedd4.2s. Our findings suggest a model in which the NC domain recruits a cellular Bro1-containing protein (s) via its N-terminal region, and possibly an additional cellular factor(s) through its C-terminal portion, which cooperate with the PTAP motif in the adjacent p6 domain to facilitate HIV-1 release (Figure 10B).

In summary, our studies present evidence strongly supporting a model in which the NC domain of Gag cooperates with L domain motifs in the adjacent p6 domain to facilitate HIV-1 release. We find that the NC domain of Gag is the site that interacts with the Bro1 domain of Alix to recruit ESCRT-III CHMP4 proteins that are necessary for HIV-1 release, thus providing a more complete model for HIV-1 budding mediated via the LYPX_n_L/Alix pathway. Additionally, we demonstrate that the NC domain of Gag plays a key role in HIV-1 release via the PTAP/Tsg101 pathway. However, although our findings favor a model in which NC promotes viral release by recruiting a Bro1-containing protein, and possibly additional host factors, more detailed mechanistic studies of the role of NC in HIV-1 budding are clearly warranted.

## Materials and Methods

### Proviral constructs

The wild-type (wt) full-length HIV-1 molecular clone pNL4-3 and the mutant pNL4-3 PTAP- (gift from Eric Freed) were previously described [11],[80]. The pNL4-3 DelNC/PR- and pNL4-3 PR- plasmids (both gifts from David Ott) contain a deletion of NC from residue 5 to 52 and/or a G75R mutation in the protease sequence, respectively [62]. The pNL4-3 SSHS/SSHS construct (gift from Robert Gorelick, called 2Zf- in the text) has all cysteine (C) residues of the nucleocapsid (NC) mutated to serine (S) [73]. The _23_YP_24_ to _23_LV_24_ mutation in Gag p6 was introduced by site directed mutagenesis of pNL4-3 wt and pNL4-3 PTAP- using overlap extension PCR. The 5′ primers contained either the upstream *Sph*I or the downstream *Age*I restriction sites and the _23_LV_24_ mutation, conserving the underlying *pol* sequence. Upstream fragments amplified from pNL4-3 PTAP- also contained the PTAP to LIRL mutation. Each of the fragments was gel purified, and amplified. The full length “LV or YP-” and “PTAP-/YP-” fragments were gel purified, digested with *Sph*I/*Age*I and subcloned back into pNL4-3 using the LONG DNA ligation kit (Takara Bio Inc, Madison, Wisconsin, United States) to make pNL4-3 YP- and pNL4-3 PTAP-/YP-, respectively. The pNL4-3 RK I and RK II plasmids were constructed using the same overlap extension PCR method. In RK I, NC residues K3, R7, R10, K11, K14, K20 and K26 were substituted with alanines. RK II carries alanine substitutions of residues K26, R29, R32, K33, K34, K38, K41, K47 and R52. The PTAP-/YP- mutation in p6 was also introduced in pNL4-3 DelNC/PR-, pNL4-3 2Zf-, pNL4-3 RK I and II by a similar approach, introducing the LIRL and LV mutations simultaneously. All proviral plasmids were grown at 30°C and their integrity was checked by *HindIII* restriction digest and sequencing. Finally, the MoMLV proviral plasmid pNCA, obtained from Steve Goff, was previously described [81].

### Expression vectors

Alix/AIP-1 cDNA was kindly donated by Heinrich Göttlinger [20] and subcloned in pHM6 (Roche, Indianapolis, Indiana, United States) between *EcoR*I/*Not*I for an N-terminal HA-tagged Alix. Point mutations were introduced in Alix using the QuickChange site-directed mutagenesis kit (Stratagene, La Jolla, California, United States). Alix fragments Bro_i_ (residues 1–202), Bro1 (1–367), Bro1-V (1–702), delBroV (203–702), VPRD (364–868) and PRD (703–868) were generated by PCR amplification from the full-length cDNA templates (wt or mutant) and subcloning into pHM6 using the *EcoR*I/*Not*I restriction sites. CHMP4A-DsRed2, CHMP4C-DsRed2, EGFP-VPS4A plasmids were kindly provided by Wesley Sundquist [21]. CHMP4A and CHMP4C genes were amplified and subcloned into p3XFLAG (Sigma) between *Not*I/*Bgl*II and *Not*I/*Eco*RI respectively for N-terminally tagged CHMP4A and C. The expression vector for N-terminal FLAG-tagged CHMP4B was purchased from GeneCopoeia (Germantown, Maryland, United States). RabGAPLP/MAP cDNA (clone Image 3027777) was purchased from Open Biosystems and cloned into p3XFLAG plasmid between the *Not*I/*Eco*RI sites. The Rev-independent HIV-1 Gag-EGFP construct was kindly provided by Marilyn Resh and described previously [82]. The pGAG_wt_POL-RRE-r, pGAGΔp6POL-RRE-r constructs, as well as the pRev1 plasmid were generous gifts from Carol Carter and were described earlier [83]. HD-PTP (clone Image 6579163) cDNA was purchased from Open Biosystems and amplified to clone the full-length gene and HDBro1 fragment (residues 1–369) in pHM6 between the *Hind*III/*Eco*RI sites. Rhophilin-2 (clone Image 4830913) cDNA was purchased from Open Biosystems and amplified to clone the full-length gene and the Rho2Bro1 fragment (residues 107–507) in pHM6 between the *Kpn*I/*Not*I sites. Brox (clone image 8322687) was obtained from Open Biosystems and subcloned in pHM6 between the *Eco*RI/*Not*I sites. Nedd4.2s, which lacks the first 121 residues of Nedd4.2 was amplified from the full-length Nedd4.2 (clone ID 5528964 purchased from Open Biosystems) and subcloned in pHM6 between the *Hind*III/*Kpn*I sites. Finally, the MBP-NC construct was made by amplification of the whole NC region from pNL4-3 and cloned in pMAL-c2X (NEB, Ipswich, Massachusetts, United States) between the *Eco*RI/*Bam*HI sites. The MBP control plasmid was constructed by creating a stop codon downstream of the *Xmn*I site of the pMAL-c2X polylinker using site directed mutagenesis. All constructs were checked by restriction digest and sequencing.

### Virus-release analysis

293T cells (2.5×10^6^) were seeded into T-25 flasks and transfected the following day using Lipofectamine 2000 (Invitrogen) according to manufacturer instructions. For dominant negative (DN) experiments, a pNL4.3 to DN DNA ratio ranging from 1∶5 to 1∶10 was used. The amount of DNA was normalized for each transfection with pHM6 empty plasmid. At 24 h post-transfection, culture supernatants were filtered through 0.45 µm syringe filters and virions were pelleted at 151,000×g for 1 h on a 20% (w/v in PBS) sucrose cushion. The cells were washed once in PBS and lysed in NP-40 lysis buffer (1% [v/v] NP-40, 50 mM Tris pH 8.0, 150 mM NaCl and protease inhibitor cocktail [Complete, Roche]). Resuspended virions and cell lysis supernatants were analyzed by SDS-PAGE on 12.5% or 15% acrylamide gels and transferred to Immobilon-P membranes (Millipore, Billerica, Massachusetts, Unites States). HIV-1 Gag and p24 proteins were detected by immunoblotting using either a mouse monoclonal anti-HIV-1 p24 (NEA-9306, NEN Life Science, Boston, Massachusetts, Unites States) or an anti-HIV-1 p24 monoclonal antibody (clone 183-H12-5C available through the NIH AIDS Research and Reference Reagent Program). Protein expression was detected using a monoclonal anti-HA and anti-α-tubulin antibodies (HA-7 and DM 1A, respectively, Sigma, St. Louis, Missouri, United States). The same protocol was used for MoMLV release experiments. MoMLV p65Gag and p30CA were detected using a goat anti-p30CA antibody (generous gift from Steve Goff). HIV-1 virus release efficiency was calculated (at time 24 h post-transfection) as the ratio of virion-associated capsid (p24) to total cellular Gag (p55 + p24) both determined by densitometry analysis of western blot films using ImageJ software (Rasband, W.S., NIH, Bethesda, MD, USA, http://rsb.info.nih.gov/ij, 1997–2007). The release efficiency of NL4-3 in presence of Alix and fragments of Alix was compared to the release efficiency of NL4-3 alone which was arbitrarily set at 100%.

### Transmission electron microscopy (TEM)

293T cells were seeded at 6×10^5^/well of a 6-well plate and transfected the following day with pNL4-3 and expression vectors for HA-tagged full-length Alix or fragments using Lipofectamine 2000. At times 32 and 48 h post-transfection, the supernatants were removed and the cells were fixed for 15 min at room temperature in 2% (v/v) glutaraldehyde in 0.1 M cacodylate buffer (pH 7.4). The cells were then rinsed in cacodylate buffer and postfixed in 1% (v/v) osmium tetroxide in the same buffer. The samples were subsequently rinsed again in 0.1 N sodium acetate buffer (pH 4.2), stained in 0.5% uranyl acetate (v/v) in the same buffer, dehydrated in graded ethanol, then infiltrated overnight in pure epoxy resin. The wells were embedded in fresh resin the next day and cured at 55°C. Blocks were cut from the cured samples and mounted appropriately for ultramicrotomy. Thin sections were stained in uranyl acetate and lead citrate and stabilized by carbon evaporation. Images were obtained with a Hitachi H7600 electron microscope equipped with an AMT XL41M digital camera. Approximately 500 cells were examined for each sample and arrested budding structures attached to the cell as well as released virions were enumerated to determine the release efficiency.

### Immunoprecipitation assays

293T cells (2×10^6^) were seeded into T25 flasks and transfected the following day using Lipofectamine 2000. Forty-eight hours post-transfection, the cells were harvested, washed twice in cold PBS and lysed in RIPA buffer (0.5% NP-40, 50 mM Hepes pH 7.3, 150 mM NaCl, 2 mM EDTA, 20 mM β-glycerophosphate, 0.1 mM Na_3_VO_4_, 1 mM NaF, 1 mM PMSF, 0.5 µM DTT and protease inhibitor cocktail [Complete, Roche]). The lysates were centrifuged at 16,100×*g*, 4°C, for 10 min and supernatants were incubated at 4°C with EZview agarose beads covalently attached to either anti-HA or anti-Flag mouse monoclonal antibody (Sigma). The beads were then extensively washed in RIPA buffer prior to 2 successive elutions with the appropriate peptide (100 µg/ml). Immunoprecipitates and cell lysates (input fractions) were analyzed by SDS-PAGE and immunoblotting with anti-HA or anti-FLAG M2 antibodies (Sigma) as indicated. For HIV-1 Gag interaction studies, the same protocol was followed except that 293T cells (6×10^6^) were transfected with various expression vectors for HA-tagged or Flag-tagged proteins along with either pGAGPOL-RRE-r with pRev1, or pNL4-3 DelNCPR- plasmid. Immunoprecipitates and cell lysates (input fraction) were analyzed by SDS-PAGE and immunoblotting with anti-HA antibody (Sigma) and an HIV-positive patient serum.

### Immunofluorescence microscopy

293T cells (1.8×10^5^) were seeded on glass coverslips in 12-well plates and transfected the following day with the indicated expression vectors using Lipofectamine 2000. Twenty-four hours post-transfection, the cells were washed once in PBS, fixed for 10 min with 3.7% paraformaldehyde (EM grade, v/v in PBS), quenched in 100 mM Glycine in PBS, permeabilized with 0.5% (v/v in PBS) Triton X-100 (Sigma) for 2 min and blocked in 1% (v/v in PBS) BSA for 30 min. Primary antibody anti-HA (Sigma HA-7) was applied at 1∶5000 in 1% BSA for 1 h followed by an Alexa 633 conjugated secondary anti-mouse antibody (Invitrogen). F-actin was stained with Alexa 568 conjugated Phalloidin and nuclei were counterstained with DAPI 1 µg/ml. Coverslips were mounted with ProLong Gold (Invitrogen) on microscope glass slides and allowed to cure overnight. Sequential Z-sections (about 0.25 µm each) were obtained by confocal microscopy (Leica SP5 confocal microscope, Leica Microsystems, Exton, PA, United States). Raw data were deconvolved with Huygens Essentials software (version 3.1, Scientific Volume Imaging BV, Hilversum, The Netherlands) reconstructed in 3-D and analyzed with Imaris software (version 6.0, Bitplane AG, Zurich, Switzerland) for colocalization statistics.

### *In vitro* pull-down assay

*E.coli* TB1 (JM83 *hsdR*) cells were transformed with the pMAL control and NC plasmids, grown to exponential phase and induced with IPTG for 3 to 4 h. Cells were harvested and lysed in column buffer (20 mM Tris-HCl pH 7.5, 200 mM NaCl, 1 mM DTT, 1 mM PMSF and protease inhibitor cocktail) by sonication. Lysates were cleared by centrifugation and were incubated with amylose resin to allow binding. The resin was washed extensively with column buffer and RIPA buffer. In parallel, 293T cells were transfected with expression vectors for HA-tagged fragments of Alix as described in the Immunoprecipitation assay section. At 48 h post-transfection 293T cells were lysed in RIPA buffer and cleared lysates were incubated with immobilized MBP control or MBP-NC on resin. After extensive washes in RIPA buffer, the resin was boiled in sample buffer and analyzed by SDS-PAGE and western blot using anti-HA antibody or Coomassie blue staining.

### Accession numbers (GenBank) of genes used in this study

Alix (NM_013374), Bro1p (Yeast U37364), Brox (BC113635), CHMP4A (BC113533), CHMP4B (BC033859), CHMP4C (BC014321), HD-PTP (BC089042), Nedd4.2 (BC032597), RabGAPLP or MAP (NM_015705), Rhophilin-2 (BC036447) and VPS4A (BC047932).

## Supporting Information

Figure S1 Alix over-expression interferes with HIV-1 budding. (A) Titration of HA-Alix used in the rescue of HIV-1 PTAP- and inhibition of wt HIV-1. 293T cells were transfected with either pNL4-3 PTAP- (lanes 1–5) or pNL4-3 wt (lanes 6–9) in absence (lanes 1 & 6) or presence of 0.15 µg (lane 2), 0.7 µg (lanes 3 & 7), 1.5 µg (lanes 4 & 8) and 3 µg (lanes 5 & 9) of HA-Alix per million cells. Pelleted virions and cell lysates were analyzed by SDS-PAGE and western blot using the indicated antibodies. Note that the optimal rescue of HIV-1 PTAP- was obtained with 0.6 µg of HA-Alix (lane 3) whereas 3 µg of HA-Alix failed to rescue the release of HIV-1 PTAP- (lane 5) and inhibited wt HIV-1 (lane 9). (B) Transmission Electron Microscopy (TEM) images of thin-sectioned 293T cells transfected with the HIV-1 BH-10 molecular clone, alone (a) or with Alix (b–f) showing arrested budding particles.
(2.92 MB PDF)

Figure S2 Bro_i_ and Bro1-V over-expression interfere with HIV-1 budding. Electron micrographs of 293T cells co-transfected with pNL4-3 wt and HA-Broi (A) or Bro1-V (B) showing arrested budding particles. Arrows indicate structures arrested at late budding steps. In the inset labeled (b), an arrested particle carrying an electron-dense “ring-like” structure (arrows) is shown at a higher magnification.
(3.01 MB PDF)

Figure S3 Bro_i_ does not bind CHMP4 isoforms. 293T cells were co-transfected with HA-Alix or Alix fragments and either FLAG-tagged CHMP4A (panel A), CHMP4B (panel B), CHMP4C (panel C), or MAP/RabGAPLP (panel D). Alix and fragments were captured from cell lysates using anti-HA antibody-conjugated beads. All fractions, input and immunoprecipitated, were analyzed by SDS-PAGE and western blot using anti-HA and anti-Flag antibodies as indicated.
(2.51 MB PDF)

Figure S4 Alignment of protein sequences of Bro1 domains from five different Bro1-containing proteins. Bro1 domains from the human Alix, HD-PTP, Rhophilin-2, and Brox as well as the yeast Bro1p protein sequences were aligned using the Align X program of the Vector NTI suite. Conserved residues are highlighted in colors.
(0.14 MB PDF)

Figure S5 Bro1 domains of HD-PTP and Rhophilin-2 interact with Gag, but only Alix Bro1 promotes viral budding. (A) Schematic representation of the domain organization of Alix, HD-PTP, and Rhophilin-2 used in this experiment. PRD: Proline Rich Domain, PTP: Protein Tyrosine Phosphatase, PEST: Proline-glutamic acid (E)-Serine-Threonin rich region, RB: Rho Binding domain and PDZ: PS.D.-95, Disc-large, ZO-1 domain. (B) Bro1 domains of Alix, HD-PTP, and Rhophilin-2 co-immunoprecipitate with HIV-1 Gag. 293T cells expressing Gag-Pol alone or with HA-Bro1, HA-Bro1_I212D_, HA-HDBro1, and HA-RhoBro1 were lysed in RIPA buffer and incubated with anti-HA antibody-conjugated beads. Both input and immunoprecipitated complexes were analyzed by SDS-PAGE and western blot using indicated antibodies. (C) Over-expression of only the Alix Bro1 domain, rescued the release of the NL4-3 PTAP-/YP- virus. 293T cells were transfected with either pNL4-3 PTAP-/YP- plasmid alone or with increasing amounts of HA-Bro1, HA-HDBro1, or HA-RhoBro1. Pelleted virions and cell lysates were analyzed by SDS-PAGE and western blot using the indicated antibodies. (D) Over-expression of HA-Bro1, but not HA-HDBro1, stimulates HIV-1 release. 293T cells were transfected with either wt pNL4-3 plasmid alone or with increasing amounts of HA-Bro1 or HA-HDBro1. Pelleted virions and cell lysates were analyzed by SDS-PAGE and western blot using the indicated antibodies.
(5.82 MB PDF)
