# Supplementary figures and images for: The Nucleocapsid Region of HIV-1 Gag Cooperates with the PTAP and LYPXnL Late Domains to Recruit the Cellular Machinery Necessary for Viral Budding
Source: PLoS Pathog. 2009 Mar 13;5(3):e1000339. doi: 10.1371/journal.ppat.1000339 (PMC2651531; doi:10.1371/journal.ppat.1000339)

**A**

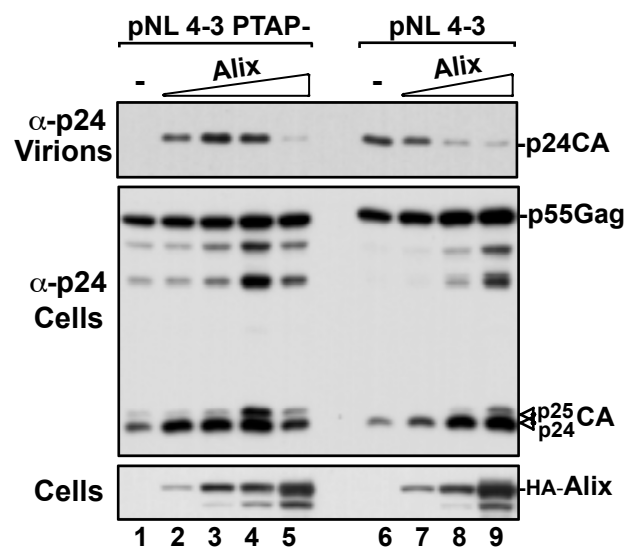

**B**

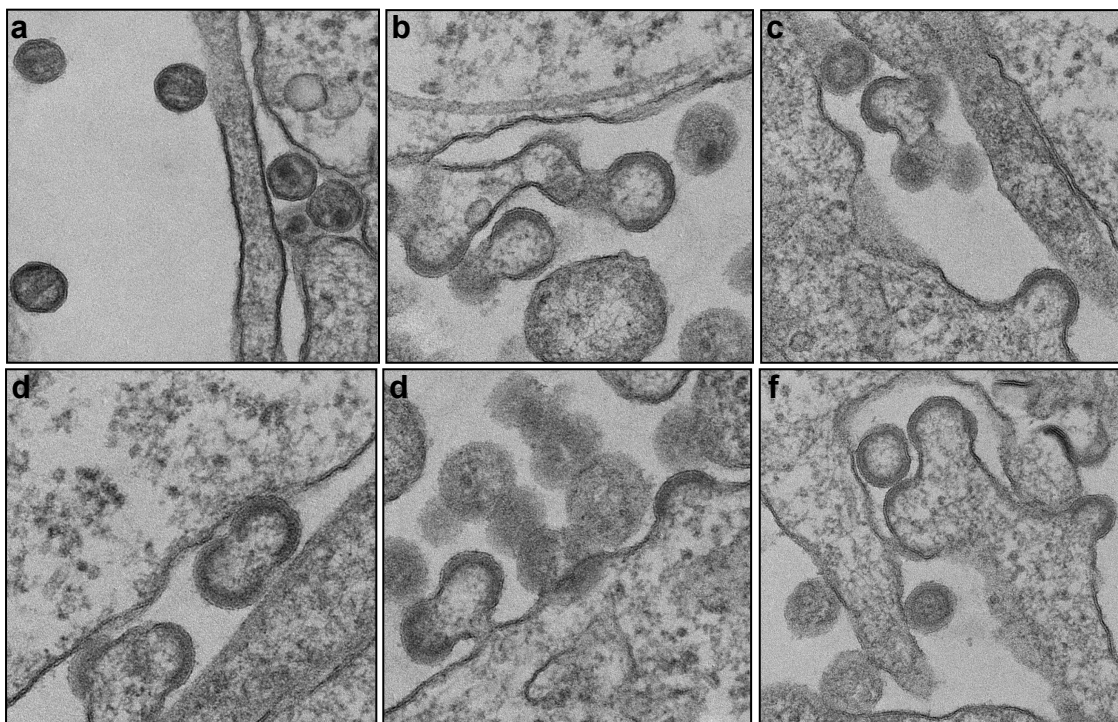

Supplement: Figure S1 — Alix over-expression interferes with HIV-1 budding. (A) Titration of HA-Alix used in the rescue of HIV-1 PTAP- and inhibition of wt HIV-1. 293T cells were transfected with either pNL4-3 PTAP- (lanes 1–5) or pNL4-3 wt (lanes 6–9) in absence (lanes 1 & 6) or presence of 0.15 µg (lane 2), 0.7 µg (lanes 3 & 7), 1.5 µg (lanes 4 & 8) and 3 µg (lanes 5 & 9) of HA-Alix per million cells. Pelleted virions and cell lysates were analyzed by SDS-PAGE and western blot using the indicated antibodies. Note that the optimal rescue of HIV-1 PTAP- was obtained with 0.6 µg of HA-Alix (lane 3) whereas 3 µg of HA-Alix failed to rescue the release of HIV-1 PTAP- (lane 5) and inhibited wt HIV-1 (lane 9). (B) Transmission Electron Microscopy (TEM) images of thin-sectioned 293T cells transfected with the HIV-1 BH-10 molecular clone, alone (a) or with Alix (b–f) showing arrested budding particles. (2.92 MB PDF) [file ppat.1000339.s001.pdf]

**A**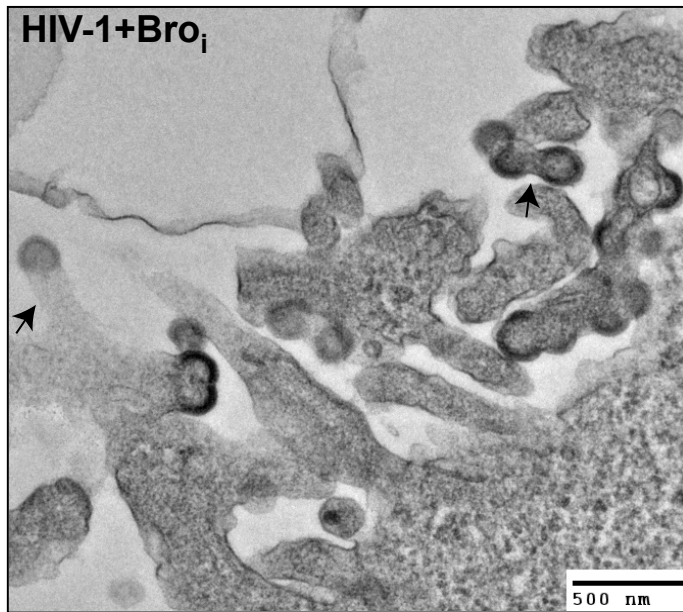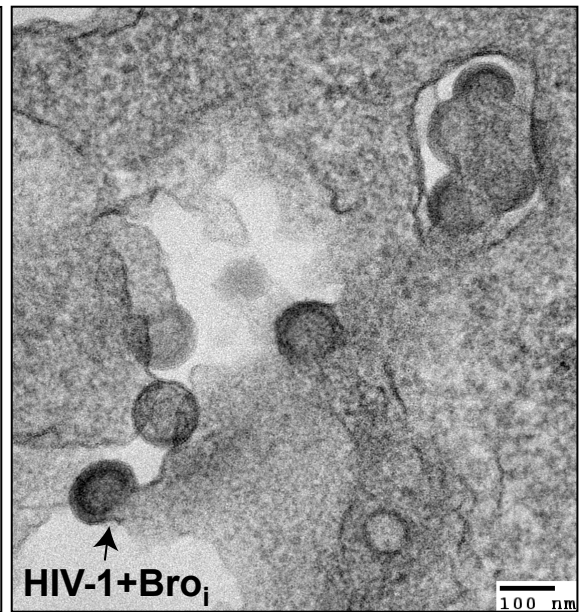**B**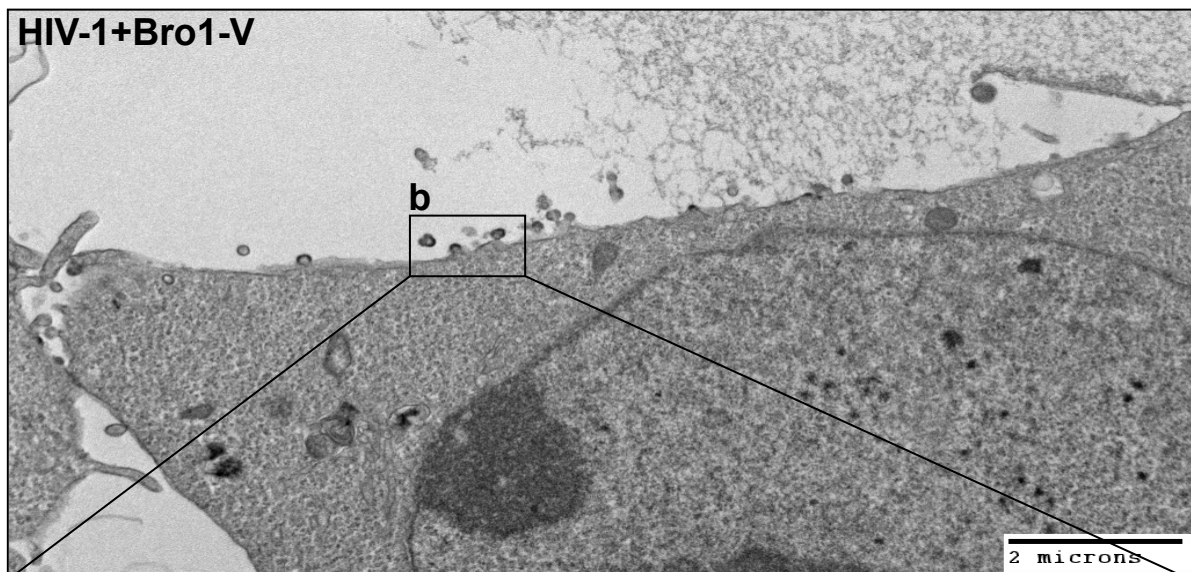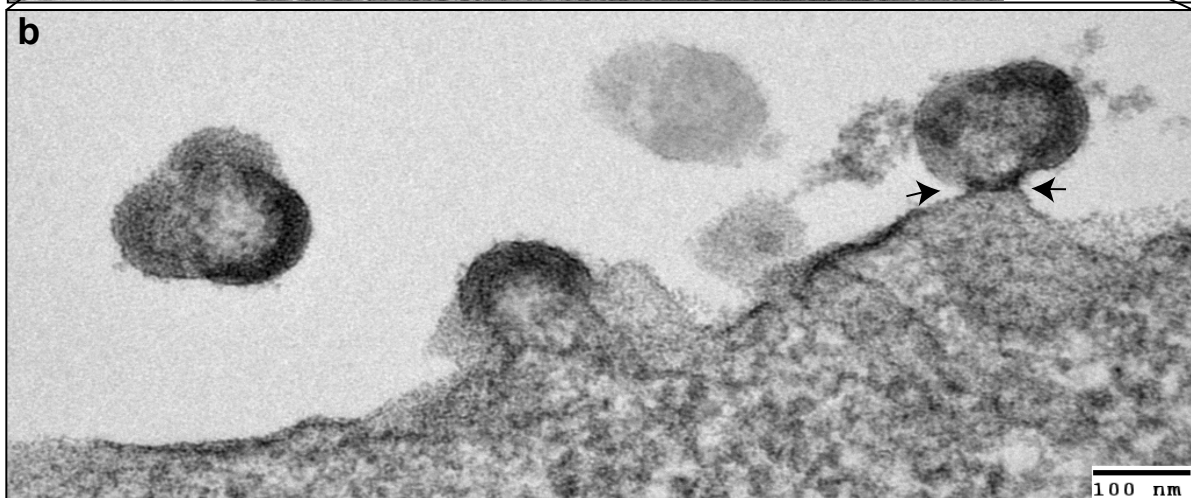

Supplement: Figure S2 — Broi and Bro1-V over-expression interfere with HIV-1 budding. Electron micrographs of 293T cells co-transfected with pNL4-3 wt and HA-Broi (A) or Bro1-V (B) showing arrested budding particles. Arrows indicate structures arrested at late budding steps. In the inset labeled (b), an arrested particle carrying an electron-dense “ring-like” structure (arrows) is shown at a higher magnification. (3.01 MB PDF) [file ppat.1000339.s002.pdf]

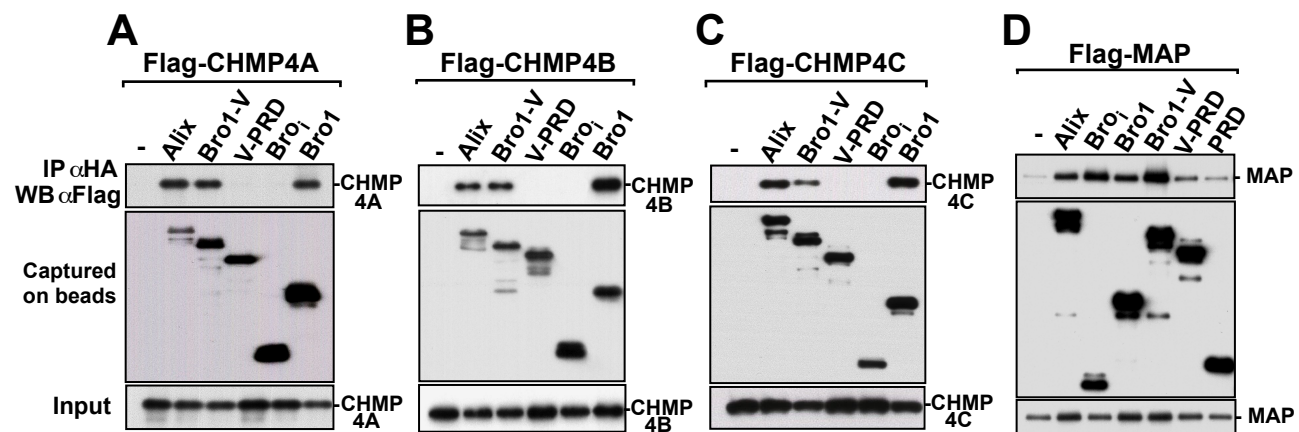

Supplement: Figure S3 — Broi does not bind CHMP4 isoforms. 293T cells were co-transfected with HA-Alix or Alix fragments and either FLAG-tagged CHMP4A (panel A), CHMP4B (panel B), CHMP4C (panel C), or MAP/RabGAPLP (panel D). Alix and fragments were captured from cell lysates using anti-HA antibody-conjugated beads. All fractions, input and immunoprecipitated, were analyzed by SDS-PAGE and western blot using anti-HA and anti-Flag antibodies as indicated. (2.51 MB PDF) [file ppat.1000339.s003.pdf]

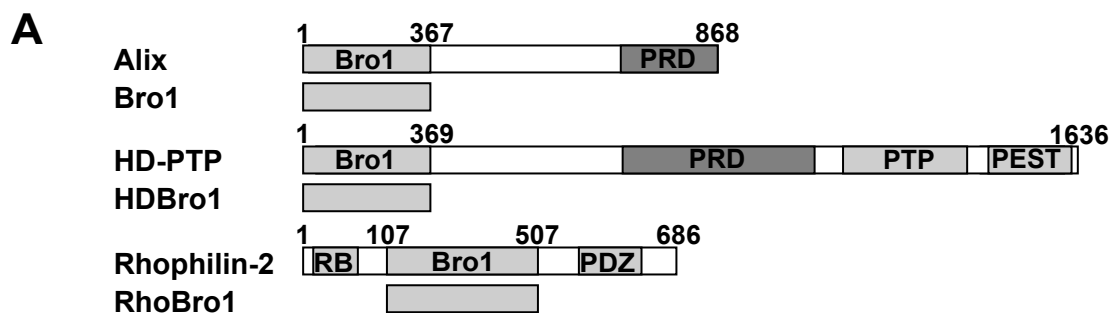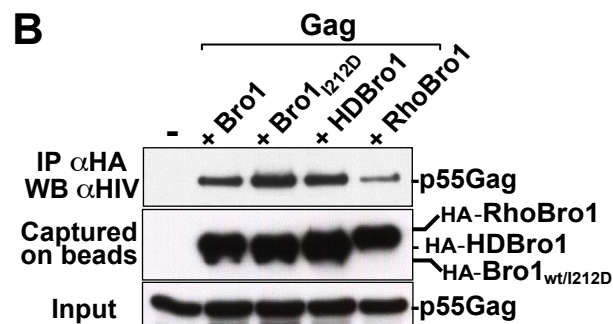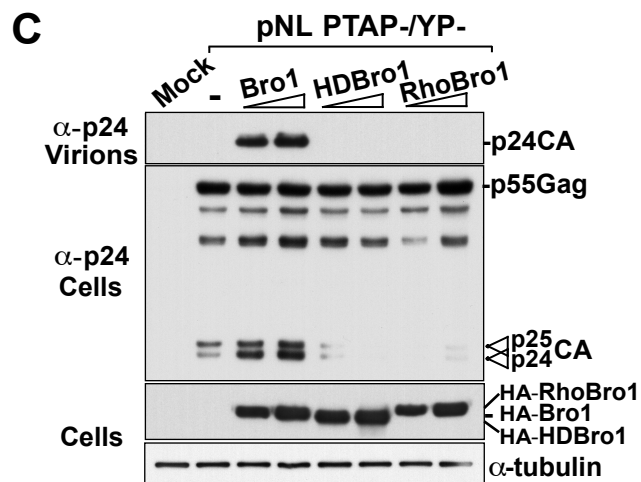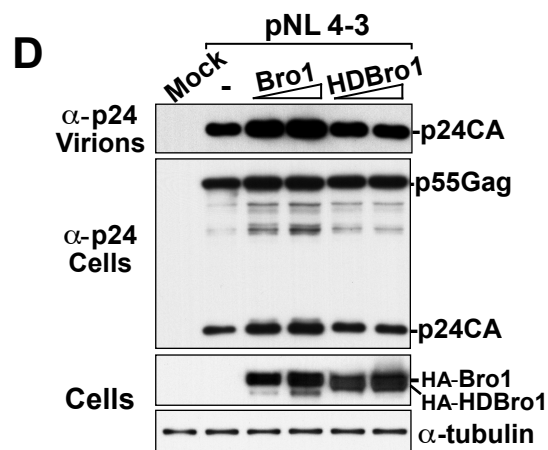

Supplement: Figure S5 — Bro1 domains of HD-PTP and Rhophilin-2 interact with Gag, but only Alix Bro1 promotes viral budding. (A) Schematic representation of the domain organization of Alix, HD-PTP, and Rhophilin-2 used in this experiment. PRD: Proline Rich Domain, PTP: Protein Tyrosine Phosphatase, PEST: Proline-glutamic acid (E)-Serine-Threonin rich region, RB: Rho Binding domain and PDZ: PS.D.-95, Disc-large, ZO-1 domain. (B) Bro1 domains of Alix, HD-PTP, and Rhophilin-2 co-immunoprecipitate with HIV-1 Gag. 293T cells expressing Gag-Pol alone or with HA-Bro1, HA-Bro1I212D, HA-HDBro1, and HA-RhoBro1 were lysed in RIPA buffer and incubated with anti-HA antibody-conjugated beads. Both input and immunoprecipitated complexes were analyzed by SDS-PAGE and western blot using indicated antibodies. (C) Over-expression of only the Alix Bro1 domain, rescued the release of the NL4-3 PTAP-/YP- virus. 293T cells were transfected with either pNL4-3 PTAP-/YP- plasmid alone or with increasing amounts of HA-Bro1, HA-HDBro1, or HA-RhoBro1. Pelleted virions and cell lysates were analyzed by SDS-PAGE and western blot using the indicated antibodies. (D) Over-expression of HA-Bro1, but not HA-HDBro1, stimulates HIV-1 release. 293T cells were transfected with either wt pNL4-3 plasmid alone or with increasing amounts of HA-Bro1 or HA-HDBro1. Pelleted virions and cell lysates were analyzed by SDS-PAGE and western blot using the indicated antibodies. (5.82 MB PDF) [file ppat.1000339.s005.pdf]
