# Supplementary material for: The Nucleocapsid Region of HIV-1 Gag Cooperates with the PTAP and LYPXnL Late Domains to Recruit the Cellular Machinery Necessary for Viral Budding
Source: PLoS Pathog. 2009 Mar 13;5(3):e1000339. doi: 10.1371/journal.ppat.1000339 (PMC2651531; doi:10.1371/journal.ppat.1000339)
Supplement: Figure S4 — Alignment of protein sequences of Bro1 domains from five different Bro1-containing proteins. Bro1 domains from the human Alix, HD-PTP, Rhophilin-2, and Brox as well as the yeast Bro1p protein sequences were aligned using the Align X program of the Vector NTI suite. Conserved residues are highlighted in colors. (0.14 MB PDF) [file ppat.1000339.s004.pdf]

```
                    (1) 1        10        20        30        40        50        60        70        80        90        100       110       120              139
Alix Human          (1) ---------MATFISVQLKKTSEVDLAKPLVKFIQQTYPSGGEEQAQYCRAAEELSKLRRAAVGRPLDKHEGALETLLRYYDQICSIEPKFPFSENQ-ICLTFTWKDAFDKGSLFGGSVKLALASLGYEKSCVLFNCAA
HD-PTP Human        (1) ----MEAVPRMPMIWLDLKEAGDFHFQPAVKKFVLKNYGENPE---AYNEELKKLELLRQNAVRVPRDFEG--CSVLRKYLGQLHYLQSRVPMGSGQEAAVPVTWTEIFSG-------KSVAHEDIKYEQACILYNLGA
Rhophilin-2 Human   (1) VYQNTEEAFTIPLIPLGLKETKDVDFAVVLKDFILEHYSEDGY---LYEDEIADLMDLRQACRTPSRDEAG--VELLMTYFIQLDFVESRFFPPTRQ-MGLLFTWYDSLTG--------VPEPAEPGAEKASVLFNTGA
Brox Human          (1) ------------MTHWFHRNPLKATAPVSFNYYGVVTGPSASKICNDLRSSRARLLELFTDLSCNPEMMKNAADSYFSLLQGFINSLDESTQESKLR-YIQNFKWTDTLQGQ------VPSAQQDAVFELISMGFNVAL
Bro1p Yeast         (1) --------MKPYLFDLKLKDTEKLDWKKGLSSYLKKSYGSSQWRTFYDEKATSELDHLRNNANGELAPSSLS--EQNLKYYSFLEHLYFRLGSKGSR-LKMDFTWYDAEYSSAQKG--LKYTQHTLAFEKSCTLFNIAV
Consensus           (1)             LI L LKET DVDF   L  FIL TY S       Y  A AEL  LRQ A   P D      E LLKYY QL  LE R      Q I L FTW DA  G       V  A  DLGFEKACVLFNIAA

                  (140) 140       150       160       170       180       190       200       210       220       230       240       250       260          278
Alix Human        (130) LASQIAAEQN---LDNDEGLKIAAKHYQFASGAFLHIKETVLSALSR--EPTVDISPDTVGTLSLIMLAQAQEVFFLKATRDKMK---DAIIAKLANQAADYFGDAFKQCQYKDT-------LPKEVFPVLAAKHCIMQ
HD-PTP Human      (124) LHSMLGAMDK---RVSEEGMKVSCTHFQCAAGAFAYLREHFPQAYS------VDMSRQILTLNVNLMLGQAQECLLEKSMLDNRK---SFLVARISAQVVDYYKEACRALENPDTASLLG-RIQKDWKKLVQMKIYYFA
Rhophilin-2 Human (126) LYTQIGTRCD---RQTEAGLESAIDAFQRAAGVLNYLKVHTFTHTP-----SYDMSPAMLSVLVKMMLAQAQECVFEKISLPGIRN-EFFMLVKVAQEAAKVGEVYQQLHQAMSQAPVKE-NIPYLWASLACVKAHHYA
                                                                 S
Brox Human        (121) WYTKYASRLAGKENITEDEAKEVHRSLKIAAGIFKHLKESHEPKLITPAEKGRDLESRLIEAYVIQCQAEAQEVTIARAIELKHA---PGLIAALAYETANFYQKADHTLSSLEP--A----YSAKWRKYLHLKMCFYT
Bro1p Yeast       (127) IFTQIAREN------INEDYKNSIANLTKAFSCFEYLSENFLN PS------VDLQSENTRFLANICHAEAQELFVLKLLNDQISSKQYTLISKLSRATCNLFQKCHDFMKEIDDDVAIYG--EPKWKTTVTCKLHFYK
Consensus         (140) LYTQIAA        TEEGLK A   FQ AAG F YLKE  L A S      VDLS  IL  LV IMLAQAQEV I KAI D IK     LIAKLA   A YF  A   L  ID        I   WK LL LKIHFY

                  (279) 279        290       300       310       320       330       340       350       360       370       380       390       400         417
Alix Human        (254) ANAEYHQSILAKQ-QKKFGEEIARLQHAAELIKTVASRYDEYVN---------------------------------VKDFSDKINRALTAAKKDNDFIYHDRVP-DLKDLDPIGKATLVKSTPVN-------------
HD-PTP Human      (250) AVAHLHMGKQAEE-QQKFGERVAYFQSALDKLNEAIKLAKGQPDTVQDA----------------------------LRFTMDVIGGKYNSAKKDNDFIYHEAVP-ALDTLQPVKGAPLVKPLPVNP------------
Rhophilin-2 Human (255) ALAHYFTAILLIDHQVKPGTDLDHQEKCLSQLYDHMPEVLTPLATLKNDQQRRQLGKSTCADHGHHEESVREASLCKKLRTIEVLQKVLCAAQERSRLTYAQHQE-EDDLLNLIDAPSVVAKTEQEVDII---------
Brox Human        (251) AYAYCYHGETLLA-SDKCGEAIRSLQEAEKLYAKAEALCKEYGETKGPGP------------------TVKPSGHLFFRKLGNLVKNTLEKCQRENGFIYFQKIPTEAPQLELKANYGLVEPIPFEFPPTSVQWTPETL
Bro1p Yeast       (252) SLSAYYHGLHLEE-ENRVGEAIAFLDFSMQQLISSLPFKTWLVEFIDFDG------------------------------FKETLEKKQKELIKDNDFIYHESVP-AVVQVDSIKALDAIKSPTWEKILEP--------
Consensus         (279) ALAHYH GI L E Q K GE IA LQ AL  L  AI      VETI                                R   DVI K L AA KDNDFIYHE VP EL  LD I A  LVK  P E
```
